# Investigating organizational resilience in a medicine and health sciences university in United Arab Emirates

**DOI:** 10.1371/journal.pone.0338728

**Published:** 2025-12-17

**Authors:** Tamara Muir, Chandra Sharma Poudyal, Romana De Lima, Farah Otaki

**Affiliations:** 1 Administrative Affairs, Mohammed Bin Rashid University of Medicine and Health Sciences, Dubai Health, Dubai, United Arab Emirates; 2 Faculty of New Media, Business, and Arts, School of Business, Southern Institute of Technology, Invercargill, New Zealand; 3 Faculty of Humanities and Business, Universal College of Learning (UCOL), Te Pūkenga, Palmerston North, New Zealand; 4 Strategy and Institutional Excellence, Mohammed Bin Rashid University of Medicine and Health Sciences, Dubai Health, Dubai, United Arab Emirates; 5 Department of Health Services Research, Care and Public Health Research Institute (CAPHRI), Faculty of Health, Medicine, and Life Sciences (FHML), Maastricht University, Maastricht, the Netherlands; King Khalid University, EGYPT

## Abstract

**Introduction:**

COVID-19 pandemic emerged in late 2019, leading to global disruption and forcing people to adapt to a new reality. The intensity of the pandemic affected many organisations’ preparedness, response, and recovery efforts, causing numerous businesses to struggle. Although no single theory fully explains why some businesses thrived during this time, the concept of organisational resilience stands out. Organisations with a resilient culture seemed better equipped to address risks, adapt effectively, and seize opportunities for innovation. Therefore, the purpose of the current study is to critically examine the response to COVID-19 of a medicine and health sciences university in Dubai, United Arab Emirates.

**Methods:**

The study relied on a convergent mixed methods approach to research. A tailor-made questionnaire was used to collect quantitative data using two 5-point Likert-type scales: ‘Opinions about Organizational Response’ and ‘Conducive Organizational Response Behaviours’ (where 110 current employees who were tenured during COVID-19 were selected, using purposive, non-probability sampling, and in turn invited to participate). Semi-structured interviews were conducted to collect qualitative data [where seven respondents who had completed the questionnaire and agreed to participate in follow-up interviews were selected (i.e., convenience, nonprobability sampling) and in turn invited to participate]. The quantitative data were descriptively and inferentially analysed. Qualitative data was analysed using an inductive six-step thematic approach. The quantitative findings were mapped onto the output of qualitative analysis using the iterative joint display analysis process.

**Results:**

A total of 70 employees completed the questionnaire (63.64%), and six out of seven invitees participated in the semi-structured interviews. The percentage of the total extent of agreement of ‘Opinions about Organizational Response’ score was 90.94%. As for the percentage of the total frequency of observation of ‘Conducive Organizational Response Behaviours’ score, it was 95.08%. The qualitative analysis generated a conceptual model, namely: ‘Enablers of Organizational Resilience’, with five interlinked themes namely: Preparedness and planning for uncertainty, Adaptation and agility, Team cohesion, Social responsibility, and Learning organisation. Four meta-inferences emerged from integrating the data findings: Response characteristics, Behaviour specificities, Consistency of opinions, and the Fundamental role of organizational culture.

**Conclusion:**

The findings reveal that organizations, in the intersect between higher education and public health, should continue on innovatively investing in agile leadership, strategic partnerships, and a robust continuous learning and development culture to better navigate future disruptions.

## Introduction

The appearance of coronavirus disease (COVID-19) in late 2019 sent shockwaves worldwide, causing global disruption, with over seven million deaths worldwide [1,2]. It affected nearly every aspect of life, from public health, the economy, travel, education, and work, forcing people to adapt to a new reality [3]. High-risk adversity such as COVID-19 pandemic, natural disasters, and economic recession crises are growing in severity and frequency [4,5].

COVID-19 is widely recognised by many economists as a ‘black swan event’ [6], it introduced ‘unknown unknowns’, which are defined as unpredictable, rare events that can have severe and widespread consequences [7]. The intensity of the pandemic impacted the preparedness, response, and recovery efforts of many organisations with some organisations surviving (some of which even thriving) while others faltered [6,8,9]. It appeared that organisations that had cultivated a resilient culture proactively addressed risks, adapted to change, and identified opportunities for innovation performed better than their peers [10,11].

While no single theory fully explains why certain businesses succeed in times of adversity and research suggesting a complex interplay of theories, ‘organisational resilience’ emerged as the most comprehensive, in terms of understanding such phenomena [4,8,12–14]. As organisations increasingly face disruption, understanding how to build and maintain resilience is essential and influences how business is done [15]. Organisational resilience (as per Duchek’s capability-based conceptualisation), which integrates concepts from risk and crisis management, may serve as a source of sustainable competitive advantage, and help explain why some organisations outperform others [13]. However, several studies [3,6,8,13] highlight a focus on immediate crisis response, with limited research conducted on understanding long-term organisational resilience and the impact on sustainable business practices from lessons learned. COVID-19 pandemic provides a unique opportunity to examine how an organisation’s crisis response (reactive) differs from proactive efforts to building capabilities that ensure sustainable competitive advantage. Organisations should learn from experience and avoid repeating past mistakes. Additionally, measuring organisational resilience remains difficult due to the lack of a standardised definition or measurement framework [9]. This underscores the need for more research to improve understanding and enhance the practical applications of organisational resilience. Accordingly, the overall purpose of the current study is to critically examine the response to COVID-19 of a medicine and health sciences university in Dubai, United Arab Emirates (UAE). The mixed methods research design adapted for the current study is meant to answer the following three questions, corresponding {as per established mixed methods article reporting standards [16]} to the quantitative, qualitative, and integration components, respectively:

1. How do employees rate the organization’s anticipatory measures, preparedness strategies, response mechanisms, and adaptive capabilities in responding to COVID-19, and what factors affected their perception?
2. What did the employees perceive to be the main factors influencing the organization’s response to COVID-19?
3. How can lessons learned from the investigated response to extreme disruption be leveraged to foster organizational resilience, within the context of the study and beyond?

## Methods

### Context of the study

Mohammed Bin Rashid University of Medicine and Health Sciences (MBRU) was set up in Dubai, UAE and in turn licensed by the Ministry of Higher Education and Scientific Research as a higher education institution towards the end of 2014 [17]. MBRU was officially established by royal decree in June 2016 [18]. MBRU is a young values-based university, characterized by a family-oriented culture of continuous learning and development, and contribution to the community-at-large (Appendix I: MBRU Inaugural Goals). MBRU currently has three colleges: College of Medicine (CoM), Hamdan Bin Mohammed College of Dental Medicine (HBMCDM), and Hind Bint Maktoum College of Nursing and Midwifery (HBMCNM), offering a range of undergraduate and postgraduate programs. MBRU’s educational programs are nationally accredited and internationally recognised [19].

As the most globalized country in the Middle East, according to an index that measures the economic, social, and political dimensions of globalisation [20–22], UAE announced the first case of COVID-19 on January 29, 2020 [1,23]. All educational activities in UAE were suspended on March 8, 2020, to slow the spread of COVID-19, which was 3 days before the World Health Organization (WHO) declared COVID-19 a pandemic [2,23]. Under directives of the Ministry of Education, all educational activities (including teaching, assessment, and administrative activities) transitioned to an online environment. MBRU resumed all operations virtually on March 22, 2020 (in 2 weeks), with all employees (faculty and staff) working remotely [24]. At the time of transition, MBRU was composed of CoM and HBMCDM only [25]. HBMCNM launched its programs in the following academic year: 2020–2021. Back then, MBRU was composed of 388 students, 61 faculty members, and 171 staff members. The rapid transition was aligned with all of UAE’s health and education regulatory policies [26].

**Fig 1:**
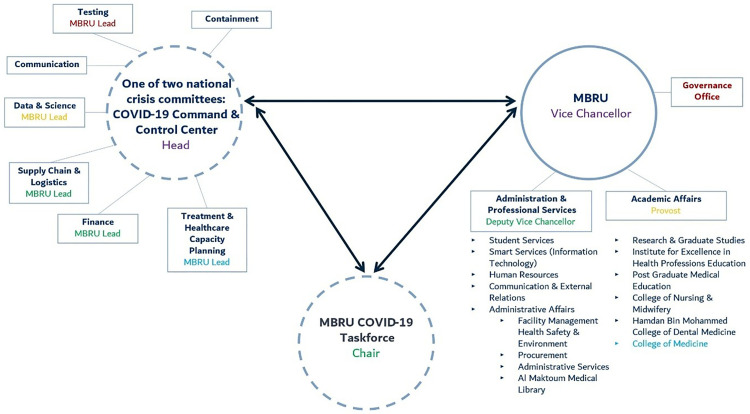
The shared governance structure between MBRU and Dubai COVID-19 Command and Control Centre, upon activation and mobilization of local capabilities in response to the pandemic. The resultant activated structure had three foci (represented as circles): maintaining learning and teaching (i.e., MBRU), while responding to the pandemic internally (i.e., university-level taskforce) and externally (i.e., Dubai COVID-19 Command and Control Centre). The dashed outlines are used to indicate the temporary activation, while the outline of the former circle is solid to signify maintenance of the university’s ongoing core activities. Using the same colour for roles is meant to represent the multiple responsibilities, across foci, that any one key stakeholder held (leading to a ‘unique’ shared governance structure).

Internally, MBRU’s response leveraged its strengths and capabilities primarily its leadership, and strong intra- and inter-organizational collaborations and strategic partnerships to enable rapid coordination, access to essential resources, and sharing of expertise. It formed a university-level COVID-19 task force, implementing work-from-home protocols, and assuring the execution of business continuity plans across all units. Scenario, preparedness, and crisis response plans were diligently completed, and a smart learning hub was created to support digital learning, leveraging proactive investment in technology. Externally, MBRU was appointed by the Dubai Government to lead the Emirate’s response to the pandemic by heading the Dubai COVID-19 Command and Control Centre, which was one of two official bodies tasked with the UAE’s response to the pandemic [27]. a platform for a whole-of-society approach to the pandemic. In other words, to the extent that the pandemic was not “a health crisis” only (it was much more than that), we needed a platform that transcends health and into all other aspects of society. Dubai COVID-19 Command and Control Centre was unique because its scope extended beyond the traditional boundaries of centres tasked with managing the operations (including responsibilities and accountabilities) of an emergency response. It provided a platform for a ‘whole-of-society’ approach to responding to the pandemic. In fact, Dubai COVID-19 Command and Control Centre pandemic did not deal with the pandemic as ‘a health-only crisis’. The platform transcended the patient and population health dimension to encapsulate all other aspects of a society; among its goals was to empower the government in striking a balance between the lives and livelihoods of its citizens. Dubai COVID-19 Command and Control Centre was headquartered at MBRU, and was tasked with setting, integrating, and implementing a roadmap for dealing with COVID-19. Accordingly, a ‘unique’ shared governance structure (Fig 1) was formed between both entities, where the Vice Chancellor of MBRU was appointed as the head of Dubai COVID-19 Command and Control Centre. Also, other MBRU leaders were assigned additional key responsibilities within Dubai COVID-19 Command and Control Centre.

Besides spearheading Dubai COVID-19 Command and Control Centre, MBRU was heavily involved in raising awareness about the pandemic through engagement with the community via multiple channels including but not limited to Dubai media, MBRU ‘Community Immunity Ambassador Program’ (reaching more than one million enrolees worldwide), and ‘Wellness on Wheels’ charity initiative. The reliability of the evidence-driven approach to operating Dubai COVID-19 Command and Control Centre and its concrete successes eventually enabled the formation of Dubai’s first integrated academic health system. In fact, MBRU, from the onset, had set out to become such a health system with a clear articulation of its intention and corresponding direction in the University’s goal upon inception [19]:

‘To advance health in the UAE and the region, through an innovative and integrated academic health system, that is nationally responsive and globally connected, serving individuals and communities.’

**Fig 2:**
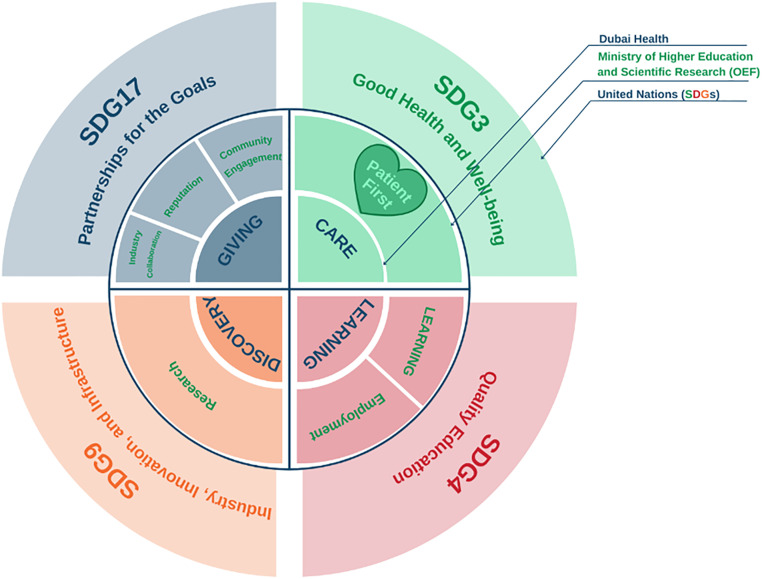
Alignment of goals (micro-, meso-, and macro-levels), as illustrated in a previously conducted mixed methods investigation that took place in the same context of the current study [32]. This figure shows how the pillars of Dubai Health (Care, Learning, and Discovery, and Giving) are feeding into the pillars of the ministerial Outcome-based Evaluation Framework (OEF) which in tun contribute to select Sustainable Development Goals (SDGs). Patient first, as the core value of Dubai Health [purposely located in the heart of the illustration, slightly towards the left (in Green, which is considered a balanced anchor for the other colours of the visible spectrum)]. This patient centricity is among the differentiators of MBRU from the rest of the Higher Education Institutions governed by the OEF of the UAE Ministry of Higher Education and Scientific Research.

Today, MBRU is the implementation vehicle of two pillars (i.e., health professions’ education/ ‘learning’ and research/ ‘discovery’ arms) of the first integrated academic health systems in Dubai, namely: Dubai Health [28] (Appendix II: Dubai Health Goals). Other than its ‘learning’ and ‘discovery’ pillars, Dubai Health oversees the operations of around 40% of the health sector in Dubai through its clinical enterprise (i.e., ‘care’ arm). The remainder of Dubai’s health sector is mainly privately owned and operated. Altogether, Dubai’s healthcare sector is regulated by the Dubai Health Authority (DHA). Besides, the ‘care’, ‘learning’, and ‘discovery’ arms, that can be mapped onto the traditional tripartite mission of the common academic health centres in North America [29], Dubai Health also has an arm related to philanthropy namely: ‘giving’. In addition to sharing its goals with Dubai Health, MBRU aligns its efforts nationally with the Ministry of Higher Education and Scientific Research- Outcome-based Evaluation Framework [30] and internationally with the United Nations- Sustainable Development Goals (SDGs) [31,32] (Fig 2).

### Research design

**Fig 3:**
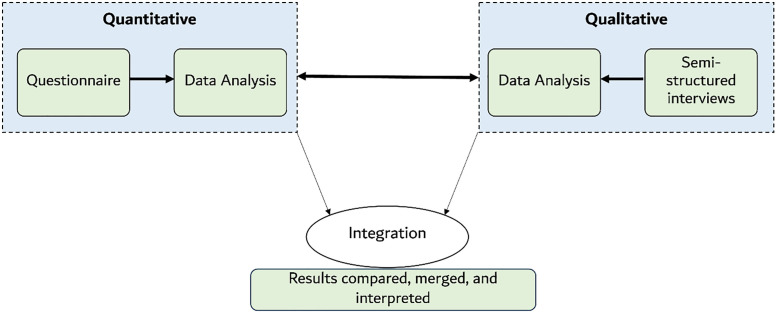
Outline of the current study’s convergent mixed methods research design.

Grounded in pragmatism and a mixed methods approach, which has been frequently commended in organizational behaviour research [33–36], this study employed a cross-sectional single case study with concurrent triangulation, also known as convergent parallel design [37] (Fig 3). Two concurrent data collection methods were integrated through a matching and connecting strategy. Data was collected on common constructs (matching), and questionnaire respondents were selected for follow-up interviews (connecting). A merging strategy entitled: joint display analysis process, compared and integrated the output of analyses [38]. This methodology allowed for the development of a comprehensive view of organisational resilience, examining ‘what’, ‘why’, and ‘how’ through integrating multiple findings as part of the efficient deployment of a mixed methods research design [37,39].

The study was approved by three relevant research governing entities: the Institutional Review Board of MBRU (MBRU IRB-2024–2) and Dubai Scientific Research and Ethics Committee (DSREC) at DHA (DSREC-SR-07/2024_07), and the Human Research Ethics Committee of the Southern Institute of Technology (SIT) (SIT Ref 2024/63). All data collection took place after obtaining the three abovementioned ethical clearances, from 5^th^ August through 8^th^ September 2024. Written informed consent (electronic and paper-based) was obtained from all participants prior to participating in the current research study. All established considerations of informed consent procedures were taken into account. As this study collects information directly from participants, ethical considerations were employed to safeguard the rights and well-being of human participants whilst contributing valuable insights into the realm of organisational resilience [37]. The participants had complete autonomy to choose whether, or not, to participate in the study. Data has been stored on a password-protected laptop for a total of five years. Participants’ and researchers’ physical and emotional well-being were protected, and potential risks were minimised. Special attention was paid to safeguarding vulnerable participants following best practices of non-maleficence [39].

### Data collection

#### Quantitative.

A tailor-made questionnaire (Appendix III: Questionnaire) was developed on Google Forms to collect primary quantitative data, using two 5-point Likert-type scales, along with participants’ demographics. The questionnaire was developed in alignment with the literature on organisational resilience, with particular emphasis on Duchek’s capability-based conceptualisation of the subject matter [8,13], where organisational resilience is believed to arise from a sophisticated interplay of a myriad of factors [4]. The purpose of this questionnaire was to capture employees’ ratings of the organization’s anticipatory measures, preparedness strategies, response mechanisms, and adaptive capabilities in responding to COVID-19, and what factors affected their perception. The 5-point Likert-type scales are widely recognised as convenient for obtaining participants’ degree of agreement with a set of statements, allowing for the generation of appropriate descriptive and inferential results [40], while web-based questionnaires are considered to offer an efficient, accessible data collection mechanism [39,41].

A data requirements table was created to ensure data relevance to the research questions and objectives. It included three data types: demographic information (including age, gender, job role, and years of experience), attitudes on organisational resilience, and behaviours and events that reflect MBRU’s long-term resilience. This approach is recommended to ensure and assure data value [39]. Google Forms provided pre-set question coding to enhance consistency, accuracy, and data collection and analysis efficiency. The questionnaire also solicited respondents’ interest to participate in follow-up semi-structured interviews, in alignment with the mixed methods research design [37,38,42].

To ensure validity and reliability, the tailormade questionnaire underwent two validation phases [39]. In the first phase, five subject matter experts from MBRU (including one faculty, one administration leader, one health and safety expert, and two researchers) reviewed the questionnaire for suitability. In the second phase, the tool was piloted with five randomly selected MBRU members to assess clarity, readability, comprehensibility, and flow. Revisions included reordering and refining questions to enhance clarity and respondent understanding.

A purposive, non-probability sampling strategy targeted the 224 employees of MBRU, focusing on the 171 employees who were tenured during COVID-19, excluding 18 who have since left the organisation. Access was obtained through workplace email lists. The sample frame was heterogeneous, including academic and administrative staff with relevant knowledge and experience during COVID-19 (N = 153). According to Qualtrics size calculations performed by Qualtrics, 110 questionnaire responses were needed to achieve a 95% confidence level with a 5% margin of error [39]. This sampling methodology is considered sufficiently efficient, is expected to provide relevant data, and is aligned with the research question and objectives [43].

The online questionnaire was disseminated on 5^th^ August 2024 using an internet-mediated strategy through Google Forms by a designated MBRU Vice President’s office member, making it accessible to respondents on their devices. Respondents received an email through workplace accounts with study information and a hyperlink for voluntary participation. According to established criteria, the questionnaire was sent at the beginning of the workweek, with follow-up emails sent after one, two, and three weeks to encourage responding [39]. The respective quantitative data collection was closed on 25^th^ August 2024. Ethical considerations were carefully followed to protect respondent rights and well-being [37,44,45]. At the beginning of the questionnaire, an information section outlined the study and detailed the respondent’s rights. The respondents were informed that completing the questionnaire and in turn clicking ‘submit’ would be equivalent to consenting to include the information that they provide in the study. Additionally, Google Forms was set not to collect identifying information, ensuring that responses remained confidential and untraceable.

#### Qualitative.

Invitations for this data collection initiative were sent on 19^th^ August 2024 to seven respondents who had completed the online questionnaire and agreed to participate in the follow-up semi-structured interviews (i.e., convenience, nonprobability sampling method). The respective qualitative data collection was completed on 8^th^ September 2024. The respective interviews balanced a structured format with the flexibility to capture participants’ varied experiences and opinions (Appendix IV: Semi-structured Interview Protocol). Five to seven participants are believed to constitute a reasonable sample size for studies using semi-structured interviews and thematic analysis, in general, and more specifically those complementing a quantitative component (such as the research design deployed for the current study) [46]. The Interview questions were developed based on a connecting strategy of the key topics outlined in the questionnaire data requirements table, following an open-ended discussion format. This ensured that the right questions were asked and enabled the triangulation of findings to develop a more comprehensive understanding of organisational resilience. This approach aimed to further understand ‘what’, ‘how’, and ‘why’ of organisational resilience at MBRU [39]. The purpose of those interviews was to explore the employees’ perception of factors influencing the organization’s response to COVID-19. Participants were contacted via their workplace email addresses, containing an interview information sheet and a consent form for the study, following a proactive approach as recommended [39]. Interview participants were explicitly asked for their voluntary participation. A statement outlining the research’s purpose, participants’ rights, and the option to withdraw from the study (including consent for audio recording) was provided. Upon returning a signed consent form, participants received a meeting invitation to facilitate the logistics of the interview process. Participants’ anonymity, confidentiality, and privacy were protected by assigning each participant a unique identifier: ‘P’ for ‘Participant’ followed by serial numbering (i.e., 1–6) followed by either ‘SL’, ‘S’, or ‘F’ for ‘Senior Leader’, ‘Staff’, or ‘Faculty’, respectively.

A third-party facilitator was engaged throughout the recruitment and interview stages to serve as an intermediary, ensuring an impartial and unbiased research process [47]. All interviews were conducted in a private room at the Al Maktoum Medical Library (AMML) on MBRU campus. Each interview lasted up to 45 minutes. Aligning with the recommendations of Saunders, Lewis, and Thornhill (2019), the interviews fostered open discussions and the externalization of rich, unbiased data [39]. Discussions were audio-recorded with the participant’s consent and transcribed verbatim using NVivo Transcription – Lumivero 2024 software [48]. The third-party facilitator reviewed the transcript while listening to the audio recording and cross-checking with interview notes to identify and correct errors, as recommended by Saunders, Lewis, and Thornhill (2019) [39]. The transcription was shared with the participants, and a brief follow-up meeting was scheduled with each of them to verify the accuracy of the collected data and clear the corresponding transcripts (i.e., member checking), as recommended by Creswell and Creswell (2023) [37]. All interview transcripts were anonymised before handing them over to the data analysers, safeguarding the study’s integrity.

### Data analysis

#### Quantitative.

The quantitative data were analysed using SPSS for Windows version 27. The descriptive analysis for the demographics’ variables, namely Gender, Age, Role, and Length of Service, revolved around calculating proportions. The descriptive analysis for the two 5-point Likert-type scales consisted of computing the overall extent of agreement score for the seven components of the ‘Opinions about Organizational Response’ tool and another overall frequency of observation score for the eight components of the ‘Conducive Organizational Response Behaviours’ tool [39,41]. The mean and standard deviation for both of those scores and each component of theirs were calculated. The reliability test of Cronbach’s Alpha and validity test of Principal Component Analysis (PCA), latter accompanied by bivariate analysis, were performed to ensure the internal consistency and external variance of the ‘Opinions about Organizational Response’ and ‘Conducive Organizational Response Behaviours’ tools [39,41].

A test of normality was conducted for those scores and each component of theirs to select the appropriate inferential analysis tests. Given that the data were all found to be not normally distributed, Mann-Whitney test was used to compare the scores and each component of theirs between female and male employees. Also, Kruskal-Wallis one-way analysis of variance test was used to assess the potentiality of associations between the following variables: ‘Opinions about Organizational Response’ and ‘Conducive Organizational Response Behaviours’ (overall scores and individual components), and Age, Role, and Length of Service. Lastly, the Bivariate Spearman Correlations was utilised to investigate the relation between the scores (and their respective components) [39,41].

#### Qualitative.

The qualitative data analysis started after the conclusion of the data collection phase. Qualitative data was analysed using an inductive six-step thematic approach based on guidelines introduced by Braun and Clarke in 2006 [49,50]. The approach was grounded in a pragmatic methodology to enable the generation of actionable insights. It was iterative, allowing for continuous refinement to ensure that the themes identified were relevant, and valuable for understanding the factors influencing the respective organization’s response to COVID-19. This analysis process was based on constructivist epistemology, using a participant-focused approach to phenomenological thematic analysis [50]. This unique interpretative approach involves the ability to identify and represent the experiences of the participants. The purpose of this approach is to understand and relate to individual participants, and their attitudes and behaviours, rather than to find casual explanations of identified variables [51]. This methodology assumes that trained qualitative data analysers can interpret individuals’ behaviours, and emotions and thoughts by comprehending self-expressions.

Prior to the analysis, the data analysers (T.M. and R.D.L.) identified personal attributes that they thought could influence their perceptions in relation to the subject under investigation. Consistency, in relation to the underlying theoretical assumptions, was maintained throughout the qualitative analysis process by one of the investigators (F.O.) [52]. By recognizing and appreciating (rather than avoiding) the data analysers’ personal involvement in the research, and by appraising interpretations according to their impact on participants, investigators, and readers [51], the quality control deployed in this investigation focused on the data analysers’ understanding rather than their scouting for the objective truths of statements. NVivo software version 12.0 plus (QSR International Pty. Ltd., Chadstone, Australia) enabled coding the data (i.e., assigning labels) and in turn classifying the text fragments tagged by the data analysers [48].

**Fig 4:**
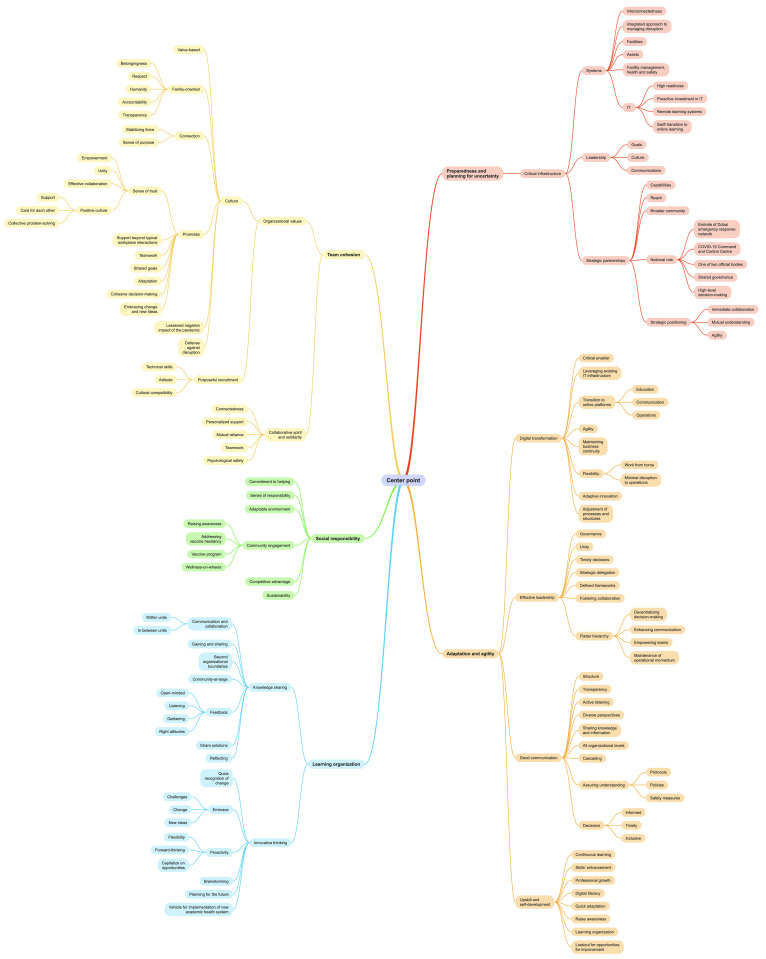
Mind map deployed as a tool to facilitate the qualitative analysis. This figure is meant to demonstrate a steppingstone in the analysis process. The output of respective analysis is represented in Fig 7.

The qualitative analysis process entailed six sequential steps: First, the data analysers, read the interview transcripts multiple times to become familiar with the data. Second, initial codes were generated and applied to characterize the content of the data. Third, recurring concepts or ideas were identified and categorised into potential themes based on relevance. Fourth, a reanalysis refined these themes, ensuring an accurate reflection of the data and incorporating any previously missed information. This refinement continued until no further valuable insights were gained (hence, data saturation was attained) (Fig 4).

Fifth, the refined themes were assigned clear, descriptive names that captured the core concepts and the study’s conceptual framework was formed. After the completion of the fifth step and prior to the sixth step (i.e., the reporting narratively on the results of the analysis), a respondent validation was conducted. The informant feedback of all the participants was obtained through a virtual meeting. One of the investigators showed the participants three PowerPoint Presentation slides. These slides included the research questions, a brief explanation of the adapted process of qualitative analysis, and the generated study’s conceptual framework. The participants were given the opportunity to share their reflections regarding the extent of resonance between their responses to the interviews and the generated conceptual framework. The meeting attendees agreed with the identified codes, and how the generated conceptual framework reflects what they had shared. Sixth, to understand what each theme represents and how the themes relate to one another, the data analysers synthesized a narrative supported by examples from the qualitative data as a means to reporting on the qualitative results. This aligns with established guidelines, including the Standards for Reporting Qualitative Research (SRQR) [16,53,54].

### Integration

The quantitative analysis findings were mapped onto the output of qualitative analysis using an integration protocol. This was done through the iterative, side-by-side joint display analysis process [34]. Furthermore, a narrative approach called weaving was used to describe the integration results thematically [55], providing a more comprehensive understanding of employees’ perceptions of organisational resilience at MBRU, including how the organization prepared, adapted, and responded to the pandemic. This approach is meant to address the limitations of individual methods, enhancing the credibility and validity of the study through a multifaceted examination of the overall research purpose. It widens the viewpoint, further enriching the study’s outcomes [38]. The integration brought together numerical findings about MBRU’s resilience with detailed insights and reflections on participants’ lived experiences.

## Results

In alignment with the guidelines of reporting on mixed methods research [16] that were adhered to for the current study, the analysis of the quantitative data addressed the first research question of the current study, while the output of qualitative analysis answered the second research question. Furthermore, as previously mentioned in the Methodology section, the third research question was addressed through the integration of quantitative and qualitative findings.

### Output of quantitative analysis

**Fig 5:**
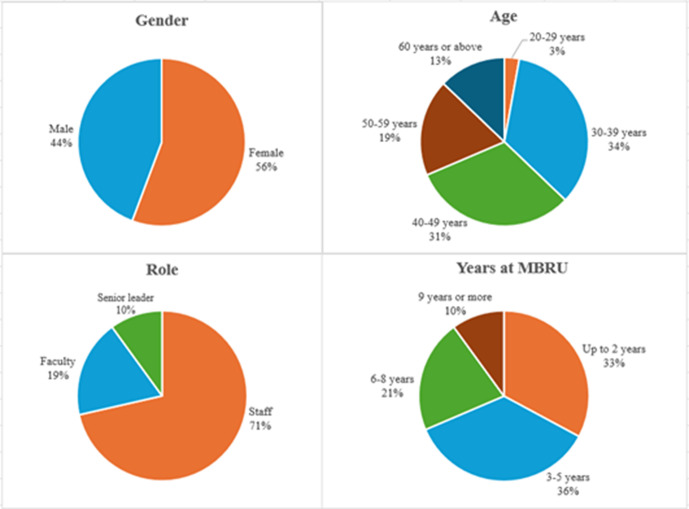
Demographic information of questionnaire respondents.

Of 110 employees invited to respond to the online questionnaire, 70 responded (63.64%). As shown in Fig 5, out of the 70 participants, 39 (55.7%) were female, and the rest were male. In terms of their ages, two (2.9%) participants were between 20 and 29 years, 24 (34.3%) were between 30 and 39 years, 22 (31.4%) were between 40 and 49 years, 13 (18.6%) were between 50 and 59 years, and nine (12.9%) were 60 years or above. As for the participant roles, 50 (71.4%) were staff, 13 (18.6%) were faculty, and seven (10%) were senior leaders. At the time of the study, 23 (32.9%) participants had been working for MBRU for up to 2 years, 25 (35.7%) participants between 3 and 5 years, 15 (21.4%) participants between 6 and 8 years, and seven (10%) for 9 years or more.

The reliability score of Cronbach’s Alpha for the ‘Opinions about Organizational Response’ tool was 91.60%. The percentage of the total extent of agreement of ‘Opinions about Organizational Response’ score was 90.94%, as per Table 1. According to the PCA (Kaiser-Meyer-Olkin Measure of Sampling Adequacy), 84% of the variance can be explained by the instrument (p < 0.001). Along the same lines, the Bivariate Spearman Correlations showed how the changes in the ‘Opinions about Organizational Response’ score can be explained by changes in all seven components (p < 0.001).

**Table 1:** Output of descriptive quantitative analysis for 5-point Likert-type scales.

Variable		Mean (SD)	Percentage of the Mean	Category
Opinions about Organizational Response	Component 1: The organization had measures in place before the pandemic that helped respond to COVID-19 pandemic.	3.86(1)	77.2%	A
	Component 2: The organization was sufficiently prepared to respond to the challenges of COVID-19 pandemic.	4.39(0.79)	87.8%	A-SA
	Component 3: The actions taken by the organization in response to COVID-19 pandemic were timely.	4.81(0.57)	96.2%	SA
	Component 4: The actions taken by the organization in response to COVID-19 pandemic were effective.	4.73(0.64)	94.6%	SA
	Component 5: The organization exhibited sufficient adaptability in handling uncertainties of COVID-19 pandemic.	4.64(0.68)	92.8%	A-SA
	Component 6: The experience gained from COVID-19 pandemic improved the organization’s ability to respond to future crises.	4.67(0.68)	93.4%	A-SA
	Component 7: The organization exhibited sufficient resilience in how it responded to (the challenges of) COVID-19 pandemic.	4.73(0.64)	94.6%	SA
	**Opinions about Organizational Response score**	**31.83(3.98)**	**90.94%**	**A-SA**
Conducive Organizational Response Behaviors	Component 1: There was effective collaboration *within* the different units at the organization.	4.74(0.63)	94.8%	SA
	Component 2: There was effective collaboration *between* the different units at the organization.	4.64(0.54)	92.8%	A-SA
	Component 3: Existing processes and procedures at the organization were successfully adjusted to meet the challenges of COVID-19 pandemic.	4.76(0.46)	95.2%	SA
	Component 4: Innovative solutions were implemented at the organization in response to COVID-19 pandemic.	4.66(0.56)	93.2%	A-SA
	Component 5: Communication was effective at the organization during COVID-19 pandemic.	4.74(0.50)	94.8%	SA
	Component 6: The organization’s leaders were supportive during COVID-19 pandemic.	4.90(0.39)	98%	SA
	Component 7: The organization’s staff demonstrated resilience to the changing circumstances of COVID-19 pandemic.	4.81(0.39)	96.2%	SA
	Component 8: The organization’s staff demonstrated their willingness to adapt to the changing circumstances of COVID-19 pandemic.	4.77(0.49)	95.4%	SA
	**Conducive Organizational Response Behaviors score**	**38.03(2.61)**	**95.08%**	**SA**

A = Agree (68.75-81.25%); SA = Strongly Agree (93.75-100%).

The reliability score of Cronbach’s Alpha for the ‘Conducive Organizational Response Behaviours’ tool was 81.90%. The percentage of the total frequency of observation of ‘Conducive Organizational Response Behaviours’ score was 95.08%, as per Table 1. According to the PCA (Kaiser-Meyer-Olkin Measure of Sampling Adequacy), 72.50% of the variance can be explained by the instrument (p < 0.001). Along the same lines, the Bivariate Spearman Correlations showed how changes in the ‘Conducive Organizational Response Behaviours’ score can be explained by changes in all eight components (p < 0.001).

There seemed to be no statistically significant association between the computed overall scores (and each component of theirs) and the demographics’ variables, namely Gender, Age, Role, and Length of Service (P > 0.05). The total extent of agreement of the ‘Opinions about Organizational Response’ score and the total frequency of observation of the ‘Conducive Organizational Response Behaviours’ score seemed to be significantly associated with each other (P < 0.05). Moreover, each of the overall scores appeared to be significantly associated with each component of the other score (P < 0.001).

### Output of qualitative analysis

**Fig 6:**
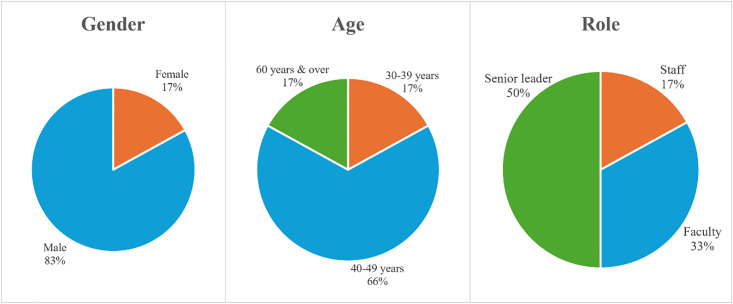
Demographic information of interview participants.

Six of the seven invited employees agreed to participate in the semi-structured interviews (85.71%). As shown in Fig 6, out of the six participants, one (17%) was female, and the rest were male. In terms of their ages, one (17%) was between 30 and 39 years, four (66%) were between 40 and 49 years, and one (17%) was 60 years or above. As for the participant roles, one (17%) was staff, two (33%) were faculty, and three (50%) were senior leaders. In the context of the study, staff refers to administrative, support, and operational personnel. Faculty refers to academic personnel, including professors, lecturers, and researchers. As for senior leaders, they refer to top management and executive team members.

**Fig 7:**
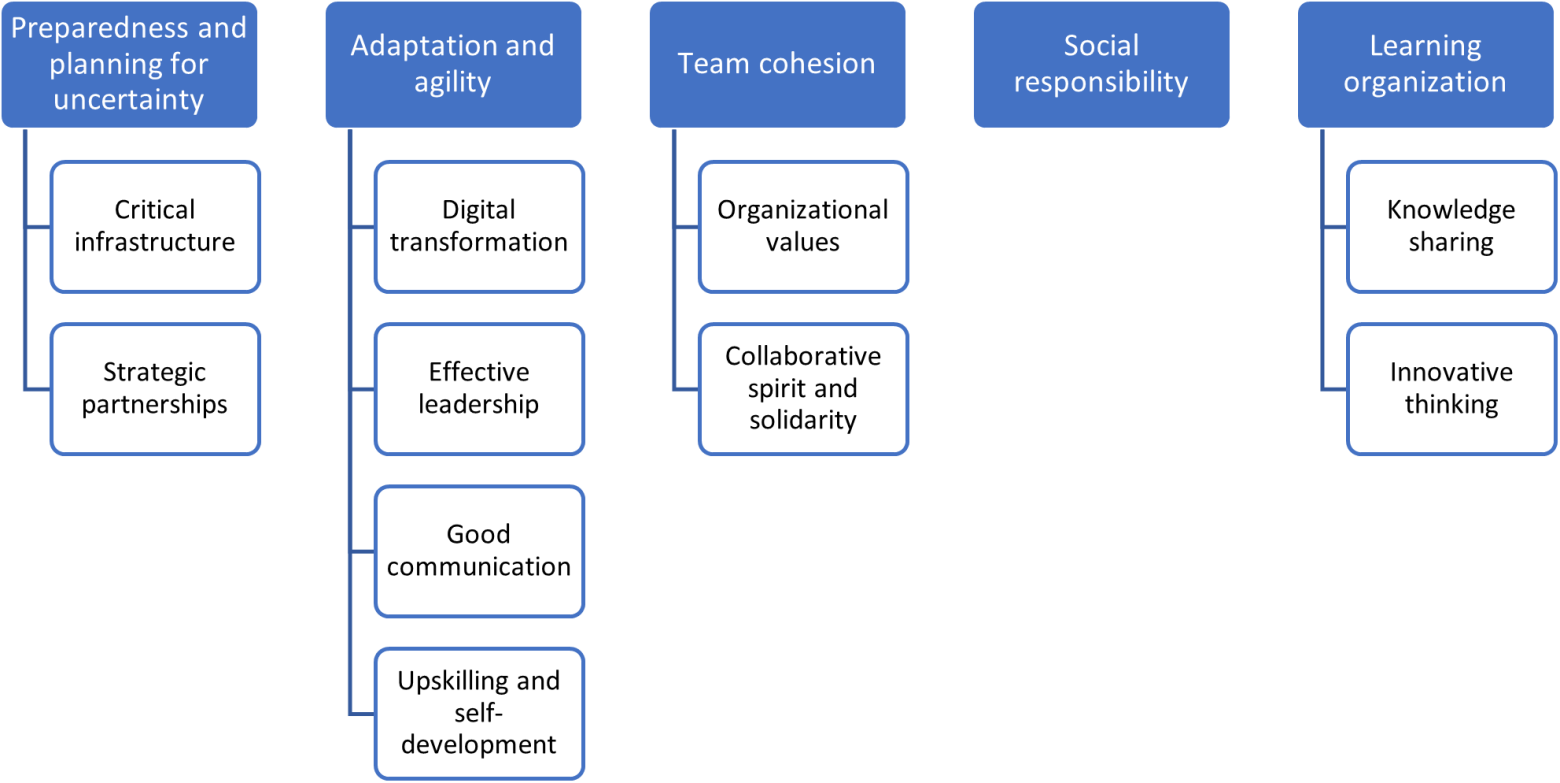
This study’s conceptual mode, namely ‘Enablers of Organizational Resilience’.

The qualitative analysis generated, as per this study’s conceptual model: ‘Enablers of Organizational Resilience’ (Fig 7), five interlinked themes namely: Preparedness and planning for uncertainty, Adaptation and agility, Team cohesion, Social responsibility, and Learning organisation. Within the Preparedness and planning for uncertainty theme, two subthemes were identified: Critical infrastructure and Strategic partners. As for the Adaption and agility theme, it includes four subthemes: Digital transformation, Effective leadership, Good communication, and Upskill and self-development. Within the Team cohesion theme, there are two subthemes: Organizational values, and Collaborative spirit and solidarity. In relation to the Social responsibility theme, it did not include any subthemes. Lastly, within the Learning organisation theme, the following subthemes were identified Knowledge sharing and Innovative thinking.

The number of participants who brought up each of the five interlinked themes are shown in Table 2.

**Table 2:** Distribution of theme mentioning.

Theme	Preparedness and planning for uncertainty	Adaptation and agility	Team cohesion	Social responsibility	Learning organization
**Number of participants**	**6**	**5**	**6**	**6**	**6**

Theme 1: Preparedness and planning for uncertainty

COVID-19 took the world by surprise. However, in some respects, MBRU was already prepared. As a young university, only five years at the onset of the pandemic, MBRU had been prioritising developing best practices for advancing health and serving its community. The University had established critical infrastructure and crisis management protocols aligned with standard practices, prior to the pandemic, enabling a rapid and effective response to the pandemic.

Information Technology (IT) systems that were already in place supported remote work and learning, and the strategic partnerships allowed MBRU to receive and provide support to adapt quickly. Six participants (n = 6) highlighted MBRU’s Preparedness and planning for uncertainty through the subthemes: Critical infrastructure and Strategic partnerships.

Subtheme 1.1: Critical infrastructure

Critical infrastructure refers to the systems, facilities, and assets vital for MBRU’s function, including facility management, health and safety protocols, IT, and communications. The robustness of these factors was essential for MBRU’s rapid and effective response to COVID-19.

Several participants stressed the importance of these systems in facilitating a seamless response to the pandemic. One senior leader (P1 SL) remarked, *“nobody was prepared for COVID-19… However, we already had remote learning systems, allowing us to switch seamlessly when the lockdown happened.”* This demonstrated how MBRU’s proactive investment in IT, allowed for a swift transition to online learning.

Another senior leader (P5 SL) commented, *“the IT infrastructure was ready... we had the tools... the network... cyber security... even before the pandemic facility management and procurement teams started ordering all the items related to the safety and making sure that the measurements are there.”* This reflected MBRU’s integrated approach to managing disruption.

A faculty participant (P6 F) echoed this, saying, “*we had fairly high readiness on the technology infrastructure.”* This underscored how technology was a central factor in MBRU’s success in transitioning online.

In the same way, faculty participant (P4 F) linked preparedness to leadership and culture, stating, *“MBRU’s strong leadership vision, organisational culture, and effective communication channels formed the foundation of preparedness... the health and safety protocols were already established, which made it easier to scale up our response to COVID-19.”* This reflected the infrastructure’s interconnectedness which contributed to MBRU’s ability to adapt quickly and effectively.

MBRU’s proactive development of critical infrastructure acted as an enabler, highlighting the importance of remaining ready against future threats or disruptions. This preparedness, in the context of the study, mitigated the pandemic’s impact, allowing MBRU to survive and even thrive.

Subtheme 1.2: Strategic partnerships

In times of crisis, strategic partnerships can significantly enhance an organisation’s capabilities, resilience, and reach. MBRU effectively leveraged its partnerships to respond swiftly to the pandemic, drawing on internal resources and its broader community. The importance of these partnerships was evident in MBRU’s appointment to lead the Emirate of Dubai emergency response network through Dubai COVID-19 Command and Control Centre.

The study participants highlighted the benefits of these partnerships. For example, a staff participant (P3 S) noted, *“the partnerships we had with government entities meant we could immediately collaborate on safety measures.”* This rapid coordination illustrates the agility that strong partnerships bring, showing that reliable relationships and mutual understanding enhance organisational resilience.

Another leader participant (P5 SL) emphasised MBRU’s national role, stating, *“MBRU played a pivotal role in the country, especially in the Dubai government. It became the Dubai COVID-19 Command and Control Centre. The Vice Chancellor of MBRU was also the head of Dubai COVID-19 Command and Control Centre.”* This illustrated how MBRU’s strategic positioning in national initiatives strengthened its crisis response, reinforcing resilience through its high-level decision-making.

MBRU’s continued development of strategic partnerships highlights that strong relationships and mutual understanding is critical for resilience as they provide access to essential resources and expertise. By leveraging its partnerships, MBRU successfully navigated the challenges presented by the pandemic, ensuring continuity while playing a leading role in Dubai’s broader COVID-19 response demonstrating how strategic collaborations enable organisations to thrive amid disruption.

Theme 2: Adaptation and agility

MBRU embraced new ways of working, and learning and teaching that, although initially seen as temporary during the pandemic, became permanent. These include hybrid learning, remote working policies, and enhanced digital transformation, supported by effective leadership, good communication, and faculty and staff upskilling. Staff resilience was central to this shift as employees needed to adapt to expanding roles and responsibilities, not only in meeting the challenges of the pandemic but also innovating towards deploying a forward-thinking approach.

Responses from five participants (n = 5) reflected MBRU’s Adaptation and agility, highlighting subthemes of Digital transformation, Effective leadership, Good communication, and Upskilling and self-development as key factors. This demonstrated MBRU’s ability to pivot quickly in reaction to changing circumstances while building a foundation for future sustainability.

Subtheme 2.1: Digital transformation

Through recognising technology as a critical enabler, MBRU swiftly leveraged its existing IT infrastructure to transition education, communication, and operations to online platforms. This agility in embracing digital transformation was central to maintaining business continuity amid COVID-19.

The study participants highlighted technology’s role in this adaptation. A senior leader (P2 SL) noted, “*the flexibility to work from home helped staff manage their responsibilities*”, ensuring minimal disruption to operations. Similarly, a senior leader (P5 SL) emphasised, *“staff quickly adapted to working from home or even ‘living online’, and balancing work, education, and family. Technology played a key role, allowing work to continue seamlessly, and even now, many of our major meetings are still held online.”* This demonstrated the seamless integration of digital tools into MBRU’s work culture, which emphasise that diverse and accessible resources, both technical and human, are vital for organisational resilience and essential for adaptation.

MBRU’s digital shift exemplifies adaptive innovation, proving that resilient organisations quickly adjust processes and structures, leveraging existing assets, competencies, and technologies. MBRU’s rapid shift to digital platforms ensured the continuity of its operations and strengthened its resilience against future disruptions by integrating digital tools into its operations.

Subtheme 2.2: Effective leadership

Effective leadership emerged as a key factor in MBRU’s ability to navigate the challenges of COVID-19, highlighting the significance of governance, timely decisions, and strategic delegation during disruption. MBRU’s leadership adopted a flatter hierarchy by ‘relative decentralization’ of decision-making, enhancement of communication, and empowerment of teams to act within defined frameworks. This approach encouraged unity, and facilitated rapid adaption and innovative solutions.

Participants emphasised the role of leadership in fostering an agile environment. For example, a senior leader (P1 SL) noted, *“the leadership team met daily to reassess… there was flexibility in arranging things… there is a provision for delegation of authority… we did not need the Vice Chancellor on-site for things to get done.”* Decentralisation allowed quick action and maintained operation momentum, where effective leadership created unity through a flexible decision-making process delegated to the experts.

A staff member (P3 S) shared a similar perspective: *“Decisions were made quickly, and leadership showed a strong will to collaborate. MBRU swiftly adapted to alternative teaching methods, fostering a positive culture of collaboration and communication.”* This showed how shared governance, where leadership empowers teams to collaborate effectively, allowed the organisation to adapt better, innovate, perform, and circumvent the challenges of disruption.

A faculty member (P4 F) added, *“leadership made decisions quickly. They were agile in their decision-making… because MBRU’s leadership were involved in the Dubai COVID-19 Command and Control Centre.”* This highlighted leadership’s increased awareness, consciousness, and agility which were recognized as key characteristics of adaptive leadership during disruption.

MBRU’s leadership demonstrated essential adaptive qualities for resilience. By ‘relative decentralisation’ of authority, fostering collaboration, and maintaining strong communication, MBRU’s leadership exemplified adaptive and agile principles.

Subtheme 2.3: Good communication

This subtheme highlights the challenge of sharing knowledge and information during the pandemic. Effective communication was crucial in MBRU’s response. The study participants emphasised the importance of clear communication at all levels of the organisation, ensuring everyone understood protocols, policies, and safety measures. This enabled quick, informed decisions and allowed strategies to adapt as new challenges emerged.

For example, a senior leader (P1 SL) remarked, *“MBRU was a growing organisation, and the communication system from the top to the different layers of leadership right up to the executive level was there… there were many channels of communication; we were able to reach out to many people to brainstorm and contribute.”* MBRU’s robust communication structure showed that diverse perspectives and inclusive decision-making strengthen resilience.

Similarly, a staff member (P3 S) noted, *“there was regular communication… we used to cascade it across the campus via our COVID-19 task force and Human Resources (HR) unit, so it was disseminated across the staff and students accordingly.”* This seamless flow across units reflected how interdependent factors, including communication and transparency, were essential for fostering organizational resilience and agility.

Agreeing with the above points, a faculty member (P4 F) noted, *“leadership always kept open communication through regular meetings… vertical and horizontal communication… access and visibility were key… people were engaged, involved.”* This highlighted how transparent communication contributed to building trust and fostering engagement and collective action. This collaborative form of leadership enhanced adaptation, innovation, and performance, where the engaged leaders constantly exchanged information and willingly listened, interacted, and communicated to enhance the organisation’s agility.

MBRU’s commitment to open, transparent, and structured communication was vital in adapting to the rapidly changing demands of COVID-19. These strategies emphasize the importance of communication in managing disruption and ensuring continuity.

Subtheme 2.4: Upskilling and self-development

This subtheme highlights the importance of continuous learning and skills’ enhancement during the pandemic (and otherwise). The rapid shift to remote working and online learning demanded rapid adaption to new digital tools, reinforcing MBRU’s resilience through a strong focus on upskilling and professional growth.

Several study participants stressed the need for digital literacy. A faculty member (P4 F) remarked, *“we were used to change, developing new programs from scratch, and quickly adapting to hybrid and online learning.”* Likewise, a senior leader (P5 SL) echoed, *“adapting online learning was a significant challenge for faculty and students, as it required a shift from the long-standing tradition of face-to-face, on-campus teaching. MBRU had to raise awareness about the importance of this transition due to the pandemic.”* The employees’ continuous learning, adaptability, and skill development were essential for building organizational resilience. Ongoing training and skill development equipped the employees to maintain operations, innovate, and address new challenges. However, one participant felt that there were opportunities for improvement in terms of HR upskilling. A faculty member (P6 F) remarked, *“I think we could do more to upskill our faculty for digital literacy.”* MBRU’s commitment to upskilling during the pandemic reflects a proactive approach to fostering organisational resilience. MBRU’s culture of continuous learning and development encourages its internal stakeholders to always lookout for opportunities for improvement.

Theme 3: Team cohesion

Team cohesion played a crucial role in MBRU’s resilience during COVID-19. The strong sense of trust, empowerment, and unity fostered effective collaboration. With a value-based, family-orientated culture, MBRU promotes trust and support beyond typical workplace interactions, enabling teams to adapt and make decisions cohesively towards shared goals. Six participants (n = 6) emphasised team cohesion through the subthemes of MBRU values, and Collaborative spirit and solidarity, underscoring the impact of culture and mutual support on resilience.

Subtheme 3.1: MBRU Values

MBRU’s organisational culture, rooted in family values, was a key driver of collective resilience. This intentional culture encouraged adaptability, lessening the negative impact of the pandemic. MBRU purposefully recruits individuals based on their technical skills, attitude, and cultural compatibility.

Several study participants highlighted the importance of these values in navigating COVID-19. A senior leader (P1 SL) shared, *“MBRU has been developed and run like a family… Most organisations do not have ‘family’ as part of what they stand for… We came to really appreciate our family-oriented culture.”* This culture of connection provided a stabilising force during the pandemic, which was anchored in a shared sense of purpose. Similarly, a staff member (P3 S) observed, *“one of the main successes during the pandemic was the strong values instilled by leadership, fostering a positive culture of collaboration and support. This culture helped us adapt to challenges and care for each other.”* This collaborative culture allowed for collective problem-solving and collective resilience.

Likewise, a senior leader (P5 SL) echoed, *“the culture here is very much like a family, it really helped us through this crisis… respect, humanity, accountability, and transparency remained at the core of all that we have been doing.”* These perspectives suggest that organisational culture acted as a defence against disruption. This showed how the social capital constituted a driver that positively impacted the social environment, fostering effective relationships, mutual understanding, and sense-making, enabling organizational resilience.

A faculty member (P4 F) also explained, *“MBRU, as a ‘greenfield university’, had a culture of adapting to change, developing programs from scratch, and altering course delivery and assessments. The overall culture, leadership vision, and open communication formed the foundation of our response… MBRU’s values, like respect and integrity, are central to its mission and vision… project management mindset was key in managing the challenges posed by COVID-19.”* This reflects how MBRU, a newly established university built from the ground up (i.e., ‘greenfield’), has fostered an adaptive culture anchored in respect, integrity, and shared vision. This allowed for the management of the challenges of the pandemic, highlighting that a culture that embraces change and new ideas is crucial to organisational resilience.

MBRU’s values-driven family culture enabled it to endure the challenges of COVID-19 and positioned it to thrive. The intentional focus on values fostered unity and belonging, inspiring teamwork and collective problem-solving. This highlights that organisational culture is a key driver for building collective resilience.

Subtheme 3.2: Collaborative spirit and solidarity

A sense of connectedness and mutual reliance promoted teamwork and organizational resilience at MBRU. The support given and received went beyond everyday social interactions, fostering close personal connections and creating a strong sense of cohesion amongst the team. This dynamic increased trust and respect within the team, allowing MBRU to adapt to the challenges of the pandemic.

Several study participants emphasised the significance of collaboration in creating an environment that is conducive to building organizational resilience. A senior leader (P1 SL) highlighted, *“collaboration was key, from the IT unit to HR, and Dubai COVID-19 Command and Control Centre… MBRU took the initiative to reach out to many people to brainstorm and contribute to take steps and make suggestions as to what can be done… everybody knows everyone.”* Emotional connections helped create high levels of communication, shaping the team and decision-making process. A faculty member (P4 F) added, *“teamwork was critical. Everyone was involved in ensuring we met our targets… we acted like a family, looking out for each other.”* This highlights the importance of the team’s connectedness in mitigating strain and enacting resilience.

A participant (P2 SL) emphasised the team’s connectedness, with support extending beyond the workplace environment: *“We held online one-on-one sessions to support team members facing emotional and mental challenges, especially those living alone. Like a family, we checked in with each other regularly, and offered help when a loved one was hospitalised or passed away.”* This sense of unity and solidarity fostered strong personal connections and mutual support. A senior leader (P5 SL) added, *“our core values are humanity, respect, and transparent accountability. We cared for our staff and community, meeting their special needs and challenges. The spirit of teamwork, passion, and collaborative effort was key to reaching those who needed help.”* These lived experiences emphasize that trust, caring relationships, authentic engagement, and psychological safety are vital for fostering a positive organisational culture and are key to building organizational resilience.

MBRU’s collaborative spirit and solidarity demonstrate that emotional connections and unity enhance teamwork and resilience. Rooted in trust and respect, these dynamics foster psychological safety and caring relationships, enabling open idea-sharing and collaboration. As teams worked cohesively, they reinforced the organisation’s overall resilience.

Theme 4: Social responsibility

MBRU consistently demonstrated a commitment to helping and supporting its community, a commitment that began before the pandemic and intensified during it. Driven by a strong sense of responsibility, MBRU’s mission of advancing health and serving humanity was embodied through organisational and individual volunteering activities. The focus on social responsibility contributed to MBRU’s resilience by fostering a supportive and adaptable environment, as noted in the responses of six participants (n = 6).

Several study participants emphasised the impact of MBRU’s community engagement during the pandemic. A senior leader (P1 SL) remarked, *“as Dubai COVID-19 Command and Control Centre… we provided education and addressed vaccine hesitancy. MBRU also reached out to the government public sector health provider, DHA, to see how they could help; many members of MBRU participated in community engagement not only at the organisational level but also for the consumers and the public people.”*

Similarly, a leader (P5 SL) reflected upon MBRU’s role in the field vaccine program, noting, *“a charity program: ‘Wellness on Wheels’, provided healthcare services free of charge for people without insurance.”* This reflects MBRU’s use of resources to meet community health needs, positioning social responsibility as an important component of organisational resilience.

A participant representing faculty (P4 F) added, *“we did Dubai media events… an online awareness initiative: ‘Community Immunity Ambassador Program’... and various media like radio show interviews.”* This helped combat misinformation and reinforced MBRU as a trusted source of knowledge and a community pillar.

MBRU’s strong emphasis on social responsibility highlights the importance of community engagement in building organizational resilience. Through active social initiatives, MBRU builds valuable stakeholder relationships, enhancing its competitive advantage and sustainability.

Theme 5: Learning organisation

During the pandemic, organisational learning was crucial for MBRU to adapt, grow, and build resilience. This involved creating, retaining, and applying knowledge to guide practice and achieve goals. Critical reflection and collective learning prepared MBRU for future challenges, with knowledge sharing and innovative thinking at the organizational core. Six participants (n = 6) highlighted MBRU’s adaptability through the subthemes of Knowledge sharing and Innovative thinking. Open communication and collaboration enabled rapid knowledge exchange, while a culture of innovation helped develop new strategies and solutions, strengthening organizational resilience.

Subtheme 5.1: Knowledge sharing

This subtheme highlighted the importance of gaining and sharing knowledge to adapt to the challenges posed by the pandemic. Participants emphasised the significance of collaboration, communication, and continuously learning from other organisations to improve their responses. By building on existing knowledge, MBRU was able to adapt to rapidly changing circumstances, enabling effective decision-making, and strengthening its resilience.

A senior leader (P2 SL) noted, *“I connected with other universities to learn about their policies and best practices. You need strong connections and teamwork to remain open-minded, to listen, and to gather feedback.”* MBRU emphasised knowledge sharing and support beyond the university’s boundaries. This approach showed how leveraging social sources, including knowledge sharing, expedites building organisational resilience.

Similarly, a staff member (P3 S) added, *“we had meetings, shared thoughts, and found solutions… we instilled this in our crisis management system in preparation to any future force majeure… we were flexible and adaptable to the crisis to curve the spread.”* This highlights the importance of reflection and learning, which is essential for adaptation, and mitigating organisational disruption and harm.

Another faculty member (P6 F) observed, *“units shifted to a cross-departmental mindset, with staff from various areas collaborating to quickly transition programs to a fully online format… still being used today.”* This highlights MBRU’s collective learning approach, shifting the focus from the individual to the collective to expedite the generation of new, relevant knowledge and the behavioural adaptation.

Overall, knowledge sharing was essential for MBRU in overcoming the challenges presented by COVID-19. By actively seeking and sharing information, MBRU was able to adapt quickly and fostered a culture of continuous learning, reinforcing resilience and establishing a framework for the future. This approach shows that information sharing, shared objectives, and strong cooperation are essential for building organizational resilience.

Subtheme 5.2: Innovative thinking

This subtheme emerged as an important component of MBRU’s learning strategy. MBRU embraced change and new ideas and took advantage of opportunities presented by the pandemic to maintain business continuity and pivot business to ensure sustainability. Participants highlighted proactive initiatives, flexibility, and forward-thinking strategies that allowed MBRU to adapt to evolving circumstances, while creating and capitalizing upon new opportunities.

A senior leader (P1 SL) remarked, *“they were able to brainstorm, take steps, and make suggestions as to what can be done… the education and examination system was modified to deliver practical teaching and examination using alternative methods.”* Similarly, a faculty member (P4 F) stated, *“MBRU is planning for the future… it was as innovative as possible, responding to changing circumstances.”* MBRU demonstrated its ability to proactively adapt and recognise change more quickly than other universities. This highlighted the importance of anticipation capabilities. By continuously fostering new ways of working and encouraging innovative ideas, MBRU was well-prepared to address the challenges of disruption and seize unexpected opportunities.

A senior leader (P5 SL) shared, *“MBRU played a pivotal role in the country, serving as Dubai COVID-19 Command and Control Centre for managing COVID-19 response… MBRU is now a key part of Dubai Health, the newly formed integrated academic health system.”* This role enabled MBRU to support national strategies while also innovating at an organizational level. Cultivating such an innovative culture was a crucial factor for anticipating and adapting to challenges and building resilience; it enabled MBRU to effectively respond, and capitalize on unexpected disruptions to grow and prosper.

Furthermore, faculty member (P6 F) highlighted, *“MBRU shifted the organisational model and trusted its mid-management and staff to deliver its objectives… MBRU showcased its capabilities… it led to MBRU becoming the vehicle for implementation of the newly established integrated academic health system.”*

Since its inception, MBRU leadership envisioned forming an integrated academic health system in Dubai modelled on international best practices. MBRU’s strong organizational response to COVID-19, along with MBRU leadership’s national role in Dubai COVID-19 Command and Control Centre provided an opportunity to present the concept of an ‘integrated academic health system’ to governmental stakeholders. Ultimately, this vision received approval, and MBRU assumed key leadership positions to govern the initiative. As a result, MBRU now leads the learning and discovery pillars of the newly established academic health system. The success of this initiative demonstrates MBRU’s resilience and sustainability, showcasing its ability to navigate disruption while creating and seizing opportunities for long-term growth. This proves that creative responses to disruption and new opportunities can be achieved by continuously stimulating innovative ideas and novel working methods.

### Output of integration

Four meta-inferences emerged from integrating the data findings, as illustrated in Table 3: Response characteristics, Behaviour specificities, Consistency of opinions, and the Fundamental role of organizational culture. Firstly, the quantitative analysis showed that the employees agreed that MBRU effectively responded. Similarly, qualitatively, the analysis uncovered what the employees perceived as the underlying characteristics of the effective responses. This is how the integration *enhanced* the understanding of the response characteristics. Secondly, the quantitative findings showed that the employees observed conducive organizational behaviours as part of the response, while the qualitative analysis described those behaviours. This meta-inference also *enhanced* the understanding of behavioural specificities. Thirdly, the quantitative and qualitative analyses showed the consistency of opinions across employees and are hence considered to have generated *aligned* findings. Lastly, the inferential quantitative analysis showed that the more the employees agreed with the effectiveness of the organizational response, the more they perceived organizational behaviour to be conducive. The qualitative analysis, however, showed that the organizational culture is (actually) what drives the conducive behaviours, and as such, it *refined* the overall understanding of the subject matter.

**Table 3:** The iterative side-by-side joint display analysis process (of integrating the data findings) resulted in four meta-inferences: Response characteristics, Behaviour specificities, Consistency of opinions, and the Fundamental role of organizational culture. The secondary colour Green emerged by mixing the primary colour Blue with the primary colour Yellow (symbolising the critical thinking that took place to generate the meta-inferences from the integration of two sets of primary inferences). The integration led to enhancement, alignment, or refinement of the researchers’ overall understanding of the subject matter.

Quantitative 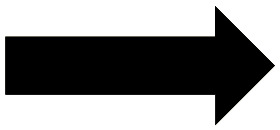	Meta-inferences	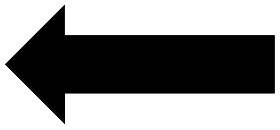 Qualitative
Employees agreed that MBRU effectively responded to COVID-19	**Response characteristics** *(Enhancement)*	Employees explained that MBRU’s effective response to COVID-19 was driven by preparedness, adaptability, team cohesion, social responsibility and learning
Employees observed conducive organizational behaviors at MBRU in response to COVID-19	**Behavioral specificities** *(Enhancement)*	Employees described conducive behavior themes, including preparedness, adaptability and agility, team cohesion, social responsibility and learning as critical to MBRU’s effective response to COVID-19
Employee demographics (i.e., age, gender, role, and years of experience) did not influence perceptions about MBRU’s response to COVID-19	**Consistency of opinions** *(Alignment* ***)***	There appeared to be an agreement across the employees in terms of positively appraising MBRU’s response to COVID-19
The more the employees agreed with the effectiveness of the organizational response, the more they perceived the organizational behaviors to be conducive	**Fundamental role of organizational culture** *(Refinement)*	Employees emphasized the role of the institutional culture in reinforcing MBRU’s adaptive capabilities and capacity to innovate

## Discussion

The current study revealed the employees’ perception of the response to COVID-19 pandemic of a young, innovative, family-oriented university of medicine and health sciences in Dubai, UAE. The entailed organizational analysis uncovered what the employees indicated as underlying characteristics of the organization’s effective response during the pandemic. It highlighted that the respective university’s established critical infrastructure and strategic partnerships played a key role in its resilience. The findings align with previous research that highlights three essential stages in building organizational resilience: anticipation, coping, and adaptation [6,12–14]. The current study, through its inductive qualitative analysis, also introduced a novel conceptual framework, namely: ‘Enablers of Organizational Resilience’, that can be deployed by other academic health systems, in general, and more specifically medicine and health sciences universities to proactively build their organizational resilience and preparedness for potential future disruptions. It highlights the key success factors in the MBRU’s experience with responding to COVID-19, and how these factors can be proactively fostered and deployed by other similar organizations to proactively build their resilience and innovatively thriving amid crises. Relevantly, the study introduced two quantitative data collection tools (5-point Likert-type scale): ‘Opinions about Organizational Response’ and ‘Conducive Organizational Response Behaviours’, which proved to be reliable and valid in the context of the study. These tools can be used by other similar institutions to capture their employees’ perception of the response of their organization, as a whole, and of particular conducive organizational behaviours in the face of adversity.

The current study showed that organizational preparedness is independent from the onset of crises. Ideally, it is an ongoing process of balancing preventative and responsive measures, that increases the likelihood of a successful result [56]. The corresponding framework needs to operate in a dormant (or latent) state and gets activated seamlessly in response to disruption, mobilizing internal capabilities/ capacities. Accordingly, organizations would adapt their strategies in response to the evolving challenges of disruptions. These dormant set-ups can be informed by thorough situational analyses, deploying established tools in organizational resilience {e.g., Business Continuity Management Systems [56]} or transferring ones from community-based disaster management {e.g., Vulnerability and Capacity Assessment (VCA) [57]}. This study also highlighted how proactively investing in technology, and in the development of facility and crisis management protocols enhances operational efficiency and embeds resilience into the organizational business strategies. It is established that resilient organisations take precautionary measures to ensure multiple layers of protection for all critical assets, including people, products, and properties [14]. Relevantly, MBRU’s commitment to proactive measures is evident on several fronts. For example, there are several flagship learning and teaching interventions that foster resilience, among learners, either directly or indirectly. These include a homegrown curricular course that is offered to undergraduate medical students to inspire and empower them to build their own resilience skills [58,59]. There are other interventions offered by MBRU that foster individual and collective resilience indirectly through curricular {e.g., professionalism training [60]} and co-curricular self-regulated learning. An example of a flagship co-curricular program would be the MBRU- Summer Scholars’ Program that supports the students in their journey towards becoming global citizens [61–63]. This ongoing pursuit to innovatively nurturing holistic and humanistic health professionals is in of itself evidence of the MBRU’s social responsibility which happens from the inside out. In other words, MBRU’s social responsibility stems from its family-oriented culture, and ripples outwards, through community engagement and outreach initiatives, where as the saying goes: ‘real change happens from the inside out’. Relevantly, fostering strong intra- and inter-organizational relationships and mutual understanding, as illustrated by Duchek’s capability-based conceptualisation of organizational resilience, is essential in preparing an organisation to deal with disruption as they provide additional resources, knowledge, and expertise [13,64].

The study also indicated what employees of the respective university considered as conducive organizational behaviours as part of a crisis response. It emphasized the crucial role of adaptive strategies and dynamic capabilities in effectively responding to extreme disruptions. MBRU’s rapid transition to digital platforms and flexible working policies, combined with effective leadership and communication, exemplified the adaptive approaches of resilient organisations. The literature, around the subject matter, emphasizes the importance of having a risk mindset that aligns with the organization’s unique business model and overall capacities, acknowledging there is no one-size-fits-all solution [14,65]. The study showed that MBRU’s leadership approach, characterized by forward-thinking strategies and decisive actions [66], was pivotal in adapting to the challenges of the pandemic. This aligns with established recommendations that highlight the importance of dynamic and agile leaders within an organisation. The importance of having an instigator to drive organisational evolution has been repetitively alluded to in the literature [67].

In terms of appraising the respective university’s response to the pandemic and the entailed organizational behaviours, there appeared to be consistency of opinions across all participating employees. This consistency highlights the shared perception of the organisational response and demonstrates team cohesion [68]. MBRU’s family-oriented values and culture seemed to be critical for fostering unity and cooperation. By deliberately cultivating a culture of mindfulness, prioritising trust, collaboration, and solidarity, MBRU established a strong foundation for organisational resilience. This finding is consistent with research indicating that organisations with cohesive teams, and engaged and empowered employees are better equipped to adapt to disruptions [14,64,69]. Additionally, this finding suggests a strong organisational culture built on trust and loyalty when facing change or disruption [18,33,67,70].

The study highlighted the fundamental role of organizational culture in terms of adaptive capabilities and capacity to innovate, both of which constitute a cornerstone in conducive organizational behaviours. These behaviours promote resilience, demonstrating that MBRU’s deep-rooted culture of trust, collaboration, and mindfulness drives such practices. MBRU’s learning initiatives and its proactive approach to innovation illustrate the organisation’s ability to engage in critical reflection and collaboration, which are vital for building long-term resilience and enabling organisations to achieve a strategic advantage and to respond effectively to emerging challenges [8,71]. This resonates with theories of organisational learning, which stress the importance of creating, retaining, and applying knowledge for resilience [72]. Additionally, adopting a mindset that perceives failures as opportunities allows organisations to develop enhanced capabilities and be better prepared for the future [11]. Hence, this study indicates that continuous quality enhancement and building organizational resilience go hand in hand, where MBRU’s culture is characterized by evidence-based decision-making and effective deployment of ongoing cycles of design-based research [59–63,73], where the latter constitutes a valuable opportunity to continuously improve both the practice and theory around innovative (commonly homegrown and ‘greenfield’) interventions, learning and teaching or otherwise [74–76].

A unique aspect of the respective university’s response was its commitment to prosocial behaviour and community engagement, highlighted in the theme of social responsibility, which was identified in the inductive qualitative analysis. This dual-focus, inward and outward, contributed to substantiating the organization’s resilience. The institutional commitment to add value extended beyond the institutional core mission to include outreach programs, health education, and vaccination support, all of which reinforce the notion that social responsibility is increasingly important to building organisational resilience [77,78]. The current study’s findings reinforce existing literature, showing that a multifaceted adaptive approach is essential for building resilience; it brings forth valuable insights into how the dynamic interplay of strategic planning, agile leadership, and collaborative efforts collectively enhance an organisation’s ability to survive and thrive amid disruption [79,80]. This was evident in the ‘unique’ shared governance structure that was formed as the result of the ‘relative decentralization’ of MBRU’s original organizational structure to effectively and efficiently mobilize internal resources and in turn activate Dubai COVID-19 Command and Control Centre. In alignment with local {e.g., Dubai Health- Care, Learning, and Discovery, and Giving [81]}, national {e.g., Ministry of Higher Education and Scientific Research- Outcome-based Evaluation Framework [30]}, and international strategies and agendas {e.g., United Nations- SDGs 3, 4, and 9, and 17 [31]}, MBRU has been augmenting resilience on micro-, meso-, and macro- levels [82,83], where nurturing resilient learners (including the employees of the university) has been feeding into proactively building critical organizational infrastructure. All of which has been leading to the reinforcement of the local and national response to the pandemic. The findings emphasize that resilience is not static but an emergent quality that relies on dynamic strategies tailored to each organisation’s context. Reflecting upon the organizational response reported upon in the current study, from the perspective of the established theory of organisational resilience, illustrates how these principles work in practice, contributing to a more nuanced understanding within higher education.

This study makes significant contributions in several aspects. Addressing a gap in the literature by examining long-term organisational resilience and its impact on sustainable business models, this study tapped into an area underexplored in previous studies [84]. It offers practical insights into building resilience and crisis response strategies by identifying successful approaches and lessons from MBRU’s experience, offering guidance for other organisations facing similar challenges [15]. MBRU can enhance its preparedness for future disruptions and maintain its reputation as a resilient organisation by understanding its response’s strengths and weaknesses. The proactiveness that MBRU exhibited prior to the pandemic is by virtue of its existence in Dubai: one of the world’s top global cities which stands out in its commitment to innovation and sustainable development [85,86], and not exactly in preparation for an emergency. After the unprecedented pandemic, MBRU (similar to other higher education institutions) integrated the lessons learned, strengthening its emergency preparedness to safeguard itself from future disruption [87,88]. This study’s findings contribute to broader industry knowledge [9], aiding in the development of a comprehensive understanding of complex organisational systems and their response to disruptions [89]. This can shape policy and promote innovation by creating new opportunities and approaches to mobilize organizational capabilities in the face of adversity [90]. Insights from this study help organisations strengthen resilience by prioritising investments to effectively mitigate risks. Such studies are critical to applied management, preparing managers with the knowledge, tools, and strategies to effectively lead amidst uncertainty [9].

The current study has a few limitations. The integration of data, via mixed methods research, enabled the development of a thorough, holistic understanding of the subject matter. However, the singular nature of the study might raise a concern regarding the external validity (i.e., generalisability) of the findings [39]. Correspondingly, the research investigators emphasized diverse sample selection, transparent reporting, and careful consideration of the transferability of findings to contexts similar to the one under investigation. This approach aligns with established recommendations that highlight that the richness of insights should precede broad generalisability within the selected research paradigm [41,73]. In addition, the low survey response rate may have impacted the reliability of the quantitative analysis; however, the mixed methods design effectively leveraged qualitative insights through integration, contributing to offsetting this limitation. There remains plenty of opportunities for future investigations of organizational resilience to include multiple entities, possibly across several environments to investigate potential associations of crisis response characteristics with contextual variables. Future research could also explore how resilience manifests in different organisational settings, industries, and cultural contexts, improving understanding of corresponding definitions and measurements [9]. This would likely highlight that resilience is not a one-size-fits-all solution. Also, capturing organisational resilience at a single point in time may be considered a limitation of the current study, given that the concept of organisational resilience is a multifaceted one that may take time to exhibit itself. Hence, not all aspects could be captured within this study’s limited timeframe. Furthermore, this study design was also limited in terms of establishing causalities between variables. Hence, it is recommended to build on the findings generated from this study through the deployment of longitudinal research designs to investigate the possible cause-and-effect relationships between the inductively identified enablers for organizational resilience and the crisis response characteristics. Furthermore, given today’s accelerated pace of environmental changes and the consequential increased severity of disruptions [4,26], such longitudinal research designs constitute an opportunity to observe and in turn, investigate how organisational resilience develops over time. This would further enhance the understanding and conceptualization of long-term organizational resilience as a dynamic, emergent quality rather than a fixed organizational trait.

## Conclusion

This study identified the factors that contributed to the effective response to COVID-19 of a university of medicine and health sciences, focusing on organisational resilience. It provides a critique of how the respective organisation’s preparedness strategies, adaptive capabilities, and learning processes enabled it to navigate disruption. The study’s findings identified several key drivers of the organisation’s success, including agile, proactive leadership, (strategic) partnerships, commitment to continuous learning, and cohesive organizational culture. This study also provides valuable insights into how similar institutions can prepare for and respond to disruptions. It emphasises that resilience is an emergent quality that relies on dynamic strategies tailored to the unique context of each organisation. The study contributes to a broader understanding of organisational resilience and illustrates how its principles were applied in a real-world setting. This reinforces the importance of integrating resilience into business strategies. The lessons learned from MBRU’s response highlight the significance of agility, collaboration, and innovation. Organisations must continue to refine their resilience strategies to better adapt to future challenges. This study advances theoretical knowledge and offers practical recommendations for managers and leaders seeking to build organisational resilience, in the intersect between higher education and public health or elsewhere, in an increasingly unpredictable world.

## Supporting information

S1 File Appendix I MBRU Inaugural Goals. (PDF)

S2 File Appendix II Dubai Health Goals. (PDF)

S3 File Appendix III Questionnaire. (PDF)

S4 File Appendix IV Semi-structured Interview Protocol. (PDF)

S5 File Raw Data. (XLSX)

## References

[1] WHO. Coronavirus disease (COVID-19). 2020.

[2] WHO. Pneumonia of unknown cause – China. Disease Outbreak News. 2020.

[3] Dumulescu D, Muţiu AI. Academic Leadership in the Time of COVID-19-Experiences and Perspectives. Front Psychol. 2021;12:648344. doi: 10.3389/fpsyg.2021.648344 33959076 PMC8093757

[4] Xiao L, Cao H, editors. Organizational resilience: The theoretical model and research implication. ITM Web of Conferences; 2017: EDP Sciences.

[5] Bartusevičienė I, Pazaver A, Kitada M. Building a resilient university: ensuring academic continuity—transition from face-to-face to online in the COVID-19 pandemic. WMU J Marit Affairs. 2021;20(2):151–72. doi: 10.1007/s13437-021-00239-x

[6] Gregurec I, Tomičić Furjan M, Tomičić-Pupek K. The Impact of COVID-19 on Sustainable Business Models in SMEs. Sustainability. 2021;13(3):1098. doi: 10.3390/su13031098

[7] Hajikazemi S, Ekambaram A, Andersen B, Zidane YJ-T. The Black Swan – Knowing the Unknown in Projects. Procedia - Social and Behavioral Sciences. 2016;226:184–92. doi: 10.1016/j.sbspro.2016.06.178

[8] Shaya N, Abu Khait R, Madani R, Khattak MN. Organizational Resilience of Higher Education Institutions: An Empirical Study during Covid-19 Pandemic. High Educ Policy. 2022;:1–27. doi: 10.1057/s41307-022-00272-2 35529423 PMC9063249

[9] Hillmann J, Guenther E. Organizational Resilience: A Valuable Construct for Management Research? Int J Management Reviews. 2020;23(1):7–44. doi: 10.1111/ijmr.12239

[10] Drexler MBA. 5 ways investors can create value through organizational resilience. 2023.

[11] Bradley EH, Alamo-Pastrana C. Dealing with Unexpected Crises: Organizational Resilience and Its Discontents. Adv Health Care Manag. 2022;21:10.1108/S1474-823120220000021001. doi: 10.1108/S1474-823120220000021001 36437614

[12] O’Shea M, Mou L, Xu L, Aikins R. Communicating COVID-19: Analyzing higher education institutional responses in Canada, China, and the USA. Higher Education Policy. 2022;35(3):629. 35765672 10.1057/s41307-022-00276-y PMC9226285

[13] Duchek S. Organizational resilience: a capability-based conceptualization. Bus Res. 2019;13(1):215–46. doi: 10.1007/s40685-019-0085-7

[14] D. D. Organizational Resilience: A summary of academic evidence, business insights and new thinking. 2025.

[15] Evenseth LL, Sydnes M, Gausdal AH. Building Organizational Resilience Through Organizational Learning: A Systematic Review. Front Commun. 2022;7. doi: 10.3389/fcomm.2022.837386

[16] Levitt HM, Bamberg M, Creswell JW, Frost DM, Josselson R, Suárez-Orozco C. Journal article reporting standards for qualitative primary, qualitative meta-analytic, and mixed methods research in psychology: The APA Publications and Communications Board task force report. Am Psychol. 2018;73(1):26–46. doi: 10.1037/amp0000151 29345485

[17] MBRU. Mohammed Bin Rashid University of Medicine and Health Sciences Dubai, UAE 2016 [cited 2024 5th May]. Available from: https://www.mbru.ac.ae/

[18] Du Preez L, Otaki F, Clemens T, Al-Hammadi S, Stanley A, Ho SB. A values-driven academic affiliation between a public medical school and a private healthcare provider: exploring the perceptions of key opinion leaders. 2024. 10.3389/frhs.2025.1655759 PMC12423040 40949621

[19] MBRU. Annual Report 2021-2022. Dubai, UAE: 2022.

[20] Alameddine M, Otaki F, Bou-Karroum K, Du Preez L, Loubser P, AlGurg R, et al. Patients’ and physicians’ gender and perspective on shared decision-making: A cross-sectional study from Dubai. PLoS One. 2022;17(9):e0270700. doi: 10.1371/journal.pone.0270700 36048748 PMC9436052

[21] KOF E. KOF Globalisation Index 2025 [cited 2025 1st February]. Available from: https://kof.ethz.ch/en/forecasts-and-indicators/indicators/kof-globalisation-index.html

[22] Gygli S, Haelg F, Potrafke N, Sturm J-E. The KOF Globalisation Index – revisited. Rev Int Organ. 2019;14(3):543–74. doi: 10.1007/s11558-019-09344-2

[23] Du Plessis SS, Otaki F, Zaher S, Zary N, Inuwa I, Lakhtakia R. Taking a Leap of Faith: A Study of Abruptly Transitioning an Undergraduate Medical Education Program to Distance-Learning Owing to the COVID-19 Pandemic. JMIR Med Educ. 2021;7(3):e27010. doi: 10.2196/27010 34227994 PMC8315158

[24] Otaki F, Zaher S, Du Plessis S, Lakhtakia R, Zary N, Inuwa IM. Introducing the 4Ps Model of Transitioning to Distance Learning: A convergent mixed methods study conducted during the COVID-19 pandemic. PLoS One. 2021;16(7):e0253662. doi: 10.1371/journal.pone.0253662 34264968 PMC8282011

[25] Rad FA, Otaki F, Baqain Z, Zary N, Al-Halabi M. Rapid transition to distance learning due to COVID-19: Perceptions of postgraduate dental learners and instructors. PLoS One. 2021;16(2):e0246584. doi: 10.1371/journal.pone.0246584 33556131 PMC7870061

[26] Otaki F, Amir-Rad F, Al-Halabi M, Baqain Z, Zary N. Self-reported adaptability among postgraduate dental learners and their instructors: Accelerated change induced by COVID-19. PLoS One. 2022;17(7):e0270420. doi: 10.1371/journal.pone.0270420 35834471 PMC9282474

[27] Abbas Zaher W, Ahamed F, Ganesan S, Warren K, Koshy A. COVID-19 Crisis Management: Lessons From the United Arab Emirates Leaders. Front Public Health. 2021;9:724494. doi: 10.3389/fpubh.2021.724494 34778167 PMC8585940

[28] Health D. Dubai Health 2023 [cited 2024 28th May]. Available from: https://dubaihealth.ae/

[29] Wartman S. The Transformation of academic health centers: meeting the challenges of healthcare’s changing landscape. Centers AoAH, editor. London: Academic Press, an imprint of Elsevier; 2015.

[30] Research MoHEaS. Outcome-based Evaluation Framework. In: Universities EFaRMf, editor. Abu Dhabi, United Arab Emirates: Ministry of Higher Education and Scientific Research; 2025.

[31] Nations U. The 2030 Agenda for Sustainable Development. In: Affairs DoEaS, editor. 2015.

[32] Al-Jayyousi R, Abu Mahfouz N, Otaki F, Paulus A, Czabanowska K, Zaman Q, et al. Investigating the learning value of early clinical exposure among undergraduate medical students in Dubai: a convergent mixed methods study. BMC Med Educ. 2025;25(1):638. doi: 10.1186/s12909-025-07212-9 40307797 PMC12044941

[33] Ezzeddine R, Otaki F, Darwish S, AlGurg R. Change management in higher education: A sequential mixed methods study exploring employees’ perception. PLoS One. 2023;18(7):e0289005. doi: 10.1371/journal.pone.0289005 37478071 PMC10361480

[34] Fetters MD, Guetterman TC. Development of a joint display as a mixed analysis. analysis TRrsgtmm, editor: Routledge; 2021.

[35] Cameron R, Taylor L. Mixed methods research approaches to measuring organisational culture. Handbook of Research Methods for Organisational Culture. 2022. p. 126–37.

[36] Eaves S. Mixed methods in knowledge management and organisational research. Encyclopedia of information science and technology, third edition. IGI Global. 2015. p. 623–32.

[37] Creswell JW, Plano Clark VL. Revisiting Mixed Methods Research Designs Twenty Years Later. The Sage Handbook of Mixed Methods Research Design. Sage Publications Ltd. 2023. p. 21–36. doi: 10.4135/9781529614572.n6

[38] Fetters MD, Curry LA, Creswell JW. Achieving integration in mixed methods designs-principles and practices. Health Serv Res. 2013;48(6 Pt 2):2134–56. doi: 10.1111/1475-6773.12117 24279835 PMC4097839

[39] Saunders M, Lewis P, Thornhill A, Jenkins M, Bolton D. Research Methods for Business Students. Eighth ed. Harlow, United Kingdom: Pearson Education Limited; 2019.

[40] Heo CY, Kim B, Park K, Back RM. A comparison of Best-Worst Scaling and Likert Scale methods on peer-to-peer accommodation attributes. Journal of Business Research. 2022;148:368–77. doi: 10.1016/j.jbusres.2022.04.064

[41] Saunders M. Research methods for business students. Person Education Limited; 2009.

[42] Hirose M, Creswell JW. Applying Core Quality Criteria of Mixed Methods Research to an Empirical Study. Journal of Mixed Methods Research. 2022;17(1):12–28. doi: 10.1177/15586898221086346

[43] Creswell JW, Creswell JD. Research design: Qualitative, quantitative, and mixed methods approaches. Sage Publications; 2017.

[44] Sims JM. A brief review of the Belmont report. Dimens Crit Care Nurs. 2010;29(4):173–4. doi: 10.1097/DCC.0b013e3181de9ec5 20543620

[45] Beauchamp TL. The belmont report. The Oxford textbook of clinical research ethics. 2008. p. 149–55.

[46] Guest G, Bunce A, Johnson L. How many interviews are enough? An experiment with data saturation and variability. Field Methods. 2006;18(1):59–82.

[47] Dawson C. Introduction to research methods 5th edition: A practical guide for anyone undertaking a research project. Robinson; 2019.

[48] Lumivero. NVivo 15 - The Most Trusted Qualitative Analysis Software (QDA) is Even Better 2024 [cited 2024 16th November]. Available from: https://lumivero.com/products/nvivo/

[49] Braun V, Clarke V. Using thematic analysis in psychology. Qualitative Research in Psychology. 2006;3(2):77–101. doi: 10.1191/1478088706qp063oa

[50] Kiger ME, Varpio L. Thematic analysis of qualitative data: AMEE Guide No. 131. Med Teach. 2020;42(8):846–54. doi: 10.1080/0142159X.2020.1755030 32356468

[51] Nowell LS, Norris JM, White DE, Moules NJ. Thematic analysis: striving to meet the trustworthiness criteria. International Journal of Qualitative Methods. 2017;16(1):1609406917733847.

[52] Stiles WB. Quality control in qualitative research. Clinical Psychology Review. 1993;13(6):593–618. doi: 10.1016/0272-7358(93)90048-q

[53] O’Brien BC, Harris IB, Beckman TJ, Reed DA, Cook DA. Standards for reporting qualitative research: a synthesis of recommendations. Acad Med. 2014;89(9):1245–51. doi: 10.1097/ACM.0000000000000388 24979285

[54] Dossett LA, Kaji AH, Cochran A. SRQR and COREQ Reporting Guidelines for Qualitative Studies. JAMA Surg. 2021;156(9):875–6. doi: 10.1001/jamasurg.2021.0525 33825809

[55] Fàbregues S, Molina-Azorin JF, Fetters MD. Virtual Special Issue on ‘Quality in Mixed Methods Research’. Journal of Mixed Methods Research. 2021;15:146–51.

[56] Butler C. Five steps to organisational resilience: Being adaptive and flexible during both normal operations and times of disruption. J Bus Contin Emer Plan. 2018;12(2):103–12. doi: 10.69554/njom6867 30642419

[57] Twigg J. Attitude before method: disability in vulnerability and capacity assessment. Disasters. 2014;38(3):465–82. doi: 10.1111/disa.12066 24905706

[58] Nair B, Otaki F. Promoting University Students’ Mental Health: A Systematic Literature Review Introducing the 4M-Model of Individual-Level Interventions. Front Public Health. 2021;9:699030. doi: 10.3389/fpubh.2021.699030 34249852 PMC8267876

[59] Nair B, Otaki F, Nair AF, Ho SB. Medical students’ perception of resilience and of an innovative curriculum-based resilience skills building course: A participant-focused qualitative analysis. PLoS One. 2023;18(3):e0280417. doi: 10.1371/journal.pone.0280417 36888625 PMC9994682

[60] Buhumaid R, Otaki F, Czabanowska K, Stanley A, Ezimokhai M, Jackson L, et al. Professionalism-training in undergraduate medical education in a multi-cultural, multi-ethnic setting in the Gulf Region: an exploration of reflective essays. BMC Med Educ. 2024;24(1):117. doi: 10.1186/s12909-024-05103-z 38321450 PMC10848390

[61] Senok A, John-Baptiste A-M, Al Heialy S, Naidoo N, Otaki F, Davis D. Leveraging the Added Value of Experiential Co-Curricular Programs to Humanize Medical Education. Journal of Experiential Education. 2021;45(2):172–90. doi: 10.1177/10538259211021444

[62] Otaki F, Naidoo N, Al Heialy S, John-Baptiste A-M, Davis D, Senok A. Shaping the future-ready doctor: a first-aid kit to address a gap in medical education. Int J Med Educ. 2020;11:248–9. doi: 10.5116/ijme.5fad.2d3a 33254148 PMC7883796

[63] Otaki F, Naidoo N, Al Heialy S, John-Baptiste A-M, Davis D, Senok A. Maximizing experiential co-curricular programs through Stakeholders’ Theory: An explanatory mixed methods study. Journal of Experiential Education. 2022;45(4):432–52. doi: 10.1177/10538259211073279

[64] Southwick FS, Martini BL, Charney DS, Southwick SM. Leadership and resilience. Leadership today: Practices for personal and professional performance. 2017. p. 315–33.

[65] Karunaratne D. Organizational Resilience: A Paradox-Based Conceptualization. Vidyodaya Journal of Management. 2022;8(I).

[66] Al‐Dabbagh ZS. The Role ofDecision‐makerin Crisis Management: A qualitative Study Using Grounded Theory (COVID‐19 Pandemic Crisis as A Model). J Public Affairs. 2020. doi: 10.1002/pa.2186 PMC7323116 32837321

[67] S. FG. Organisational Resilience: Building an enduring enterprise. 2015.

[68] Ramlo SE. Universities and the COVID-19 Pandemic: Comparing Views about How to Address the Financial Impact. Innov High Educ. 2021;46(6):777–93. doi: 10.1007/s10755-021-09561-x 34177079 PMC8215488

[69] Tan MZY, Prager G, McClelland A, Dark P. Healthcare resilience: a meta-narrative systematic review and synthesis of reviews. BMJ Open. 2023;13(9):e072136. doi: 10.1136/bmjopen-2023-072136 37730383 PMC10514640

[70] Liu BF, Shi D, Lim JR, Islam K, Edwards AL, Seeger M. When Crises Hit Home: How U.S. Higher Education Leaders Navigate Values During Uncertain Times. J Bus Ethics. 2022;179(2):353–68. doi: 10.1007/s10551-021-04820-5 34002104 PMC8115862

[71] Akar İ. Post-Coronavirus Disease 2019 Health Care and University: From Efficiency to Resilience. OMICS. 2020;24(9):515–7. doi: 10.1089/omi.2020.0111 32603205

[72] Harris SG. The fifth discipline: The art and practice of the learning organization, by Peter Senge, New York: Doubleday/Currency, 1990. Human Resource Management. 1990;29(3):343–8. doi: 10.1002/hrm.3930290308

[73] Kaddoura R, Faraji H, Otaki F, Radhakrishnan R, Stanley A, Paulus A, et al. High-fidelity simulation versus case-based tutorial sessions for teaching pharmacology: Convergent mixed methods research investigating undergraduate medical students’ performance and perception. PLoS One. 2024;19(8):e0302609. doi: 10.1371/journal.pone.0302609 39150900 PMC11329139

[74] Sandars J, Goh P-S. Design Thinking in Medical Education: The Key Features and Practical Application. J Med Educ Curric Dev. 2020;7:2382120520926518. doi: 10.1177/2382120520926518 32548307 PMC7273544

[75] Kimmons R CS. The Students’ Guide to Learning Design and Research. 2020. Available from: https://edtechbooks.org/studentguide?tab=search&term=

[76] Chen W, Reeves TC. Twelve tips for conducting educational design research in medical education. Med Teach. 2020;42(9):980–6. doi: 10.1080/0142159X.2019.1657231 31498719

[77] Awad J, Martín‐Rojas R. Enhancing social responsibility and resilience through entrepreneurship and digital environment. Corp Soc Responsibility Env. 2023;31(3):1688–704. doi: 10.1002/csr.2655

[78] Ma T, Wang H, Qu Y. Impact of Corporate Social Responsibility on Organizational Resilience in Construction Firms—A Study from China. Sustainability. 2024;16(19):8366. doi: 10.3390/su16198366

[79] Mao Y, Kang X, Lai Y, Yu J, Deng X, Zhai Y, et al. Authentic leadership and employee resilience during the COVID-19: The role of flow, organizational identification, and trust. Curr Psychol. 2023;:1–16. doi: 10.1007/s12144-022-04148-x 36713621 PMC9869839

[80] Klein B, Generous N, Chinazzi M, Bhadricha Z, Gunashekar R, Kori P, et al. Higher education responses to COVID-19 in the United States: Evidence for the impacts of university policy. PLOS Digit Health. 2022;1(6):e0000065. doi: 10.1371/journal.pdig.0000065 36812533 PMC9931316

[81] Health D. Connected for you First Integrated Academic Health System in Dubai, United Arab Emirates 2021 [cited 2025 14th February]. Available from: https://dubaihealth.ae/

[82] Borg N, Naderpajouh N, Scott-Young CM, Borg J. An interdisciplinary and multi-level review of resilience to inform training of human resources for critical infrastructure. International Journal of Disaster Risk Reduction. 2022;78:103113. doi: 10.1016/j.ijdrr.2022.103113

[83] Msosa WH. Records management practices at the northern region water board, Malawi. Mzuzu University; 2022.

[84] Brammer S, Branicki L, Linnenluecke M. Disrupting Management Research? Critical Reflections on British Journal of Management COVID‐19 Research and an Agenda for the Future. British J of Management. 2022;34(1):3–15. doi: 10.1111/1467-8551.12699

[85] Al-Kodmany K. Refashioning cities in the Middle East: The case of Dubai. International Journal of High-Rise Buildings. 2024;13(1):11–32.

[86] Serafeim G, Rischbieth AM, Koh HK. Sustainability, Business, and Health. JAMA. 2020;324(2):147–8. doi: 10.1001/jama.2020.8714 32629479 PMC8587651

[87] Ahmed SA, Shehata MHK, Wells RL, Amin HAA, Atwa HSM. A Step-by-Step Guide to Managing the Educational Crisis: Lessons Learned from COVID-19 Pandemic. J Microsc Ultrastruct. 2020;8(4):193–7. doi: 10.4103/jmau.jmau_79_20 33623746 PMC7883494

[88] Ratten V, Jones P. Covid-19 and entrepreneurship education: Implications for advancing research and practice. The International Journal of Management Education. 2021;19(1):100432. doi: 10.1016/j.ijme.2020.100432

[89] Nathanial P, Van der Heyden L. Crisis management: Framework and principles with applications to CoVid-19. 2020.

[90] Linnenluecke MK. Resilience in Business and Management Research: A Review of Influential Publications and a Research Agenda. Int J Management Reviews. 2015;19(1):4–30. doi: 10.1111/ijmr.12076

