# Supplementary material for: Investigating organizational resilience in a medicine and health sciences university in United Arab Emirates
Source: PLoS One. 2025 Dec 17;20(12):e0338728. doi: 10.1371/journal.pone.0338728 (PMC12711023; doi:10.1371/journal.pone.0338728)
Supplement: S1 File — (PDF) [file pone.0338728.s001.pdf]

Vision

A global hub for innovative and integrated healthcare education and research at the service of humanity.

Mission

To advance health in the UAE and the region, through an innovative and integrated academic health system, that is nationally responsive and globally connected, serving individuals and communities.

- RESPECT
- INTEGRITY
- CONNECTIVITY
- GIVING
- EXCELLENCE
