# Supplementary material for: Investigating organizational resilience in a medicine and health sciences university in United Arab Emirates
Source: PLoS One. 2025 Dec 17;20(12):e0338728. doi: 10.1371/journal.pone.0338728 (PMC12711023; doi:10.1371/journal.pone.0338728)

Dubai Health

Vision

Together We Advance Health For Humanity

Mission

We serve to impact lives and shape the future of health through the integration of care, learning, discovery and giving

Values

- Patient First *(primary value)*
- Respect
- Excellence
- Teamwork
- Integrity
- Empathy

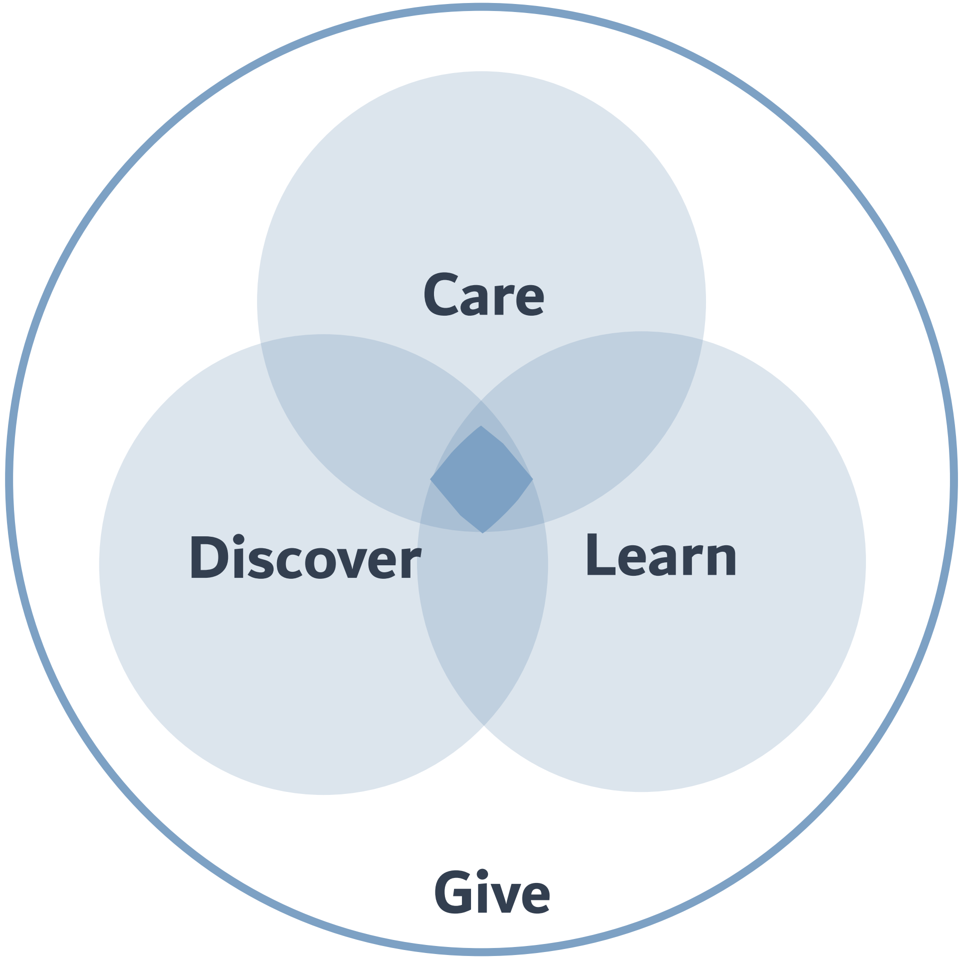

Supplement: S2 File — (PDF) [file pone.0338728.s002.pdf]
