# Supplementary material for: Investigating organizational resilience in a medicine and health sciences university in United Arab Emirates
Source: PLoS One. 2025 Dec 17;20(12):e0338728. doi: 10.1371/journal.pone.0338728 (PMC12711023; doi:10.1371/journal.pone.0338728)
Supplement: S3 File — (PDF) [file pone.0338728.s003.pdf]

Appendix: III Questionnaire

1. Please select your current age from the age range below.

   ☐20 – 29 years
   ☐30 – 39 years
   ☐40 – 49 years
   ☐50 – 59 years
   ☐60 years and over

2. What gender do you identify yourself with?

   ☐Male
   ☐Female
   ☐Gender Diverse
   ☐Prefer not to say

3. At the onset of the COVID-19 pandemic (March 11, 2020), what was your role at MBRU?

   ☐Staff
   ☐Faculty
   ☐Senior Leader

4. At the onset of the COVID-19 pandemic (March 11, 2020), how long had you been working at MBRU?

   ☐0 – 2 years
   ☐3 – 5 years
   ☐6 – 8 years
   ☐9 years and over

5. Opinions on how MBRU responded to the COVID-19 pandemic. Please indicate the level of agreement with each of the following statements.

	**Strongly Disagree**	**Disagree**	**Neutral**	**Agree**	**Strongly Agree**
a. MBRU had measures in place before the pandemic that helped respond to the COVID-19 pandemic.	☐	☐	☐	☐	☐
b. MBRU was sufficiently prepared to respond to the	☐	☐	☐	☐	☐

	challenges of the COVID-19 pandemic.					
c.	The actions taken by MBRU in response to the COVID-19 pandemic were timely.	☐	☐	☐	☐	☐
d.	The actions taken by MBRU in response to the COVID-19 pandemic were effective.	☐	☐	☐	☐	☐
e.	MBRU exhibited sufficient adaptability in handling uncertainties of the COVID-19 pandemic.	☐	☐	☐	☐	☐
f.	The experiences gained from the COVID-19 pandemic improved MBRU's ability to respond to future crises	☐	☐	☐	☐	☐
g.	The experiences gained from the COVID-19 pandemic have improved MBRU's ability to respond to future challenges.	☐	☐	☐	☐	☐
f.	MBRU exhibited sufficient resilience in how it responded to (the challenges of) the COVID-19 pandemic.	☐	☐	☐	☐	☐

6. Behaviors and events observed during the COVID-19 pandemic. Please indicate the level of agreement with each of the following statements.

		Never	Rarely	Sometimes	Frequently	Always
a.	There was effective collaboration within the different units at MBRU.	☐	☐	☐	☐	☐
b.	There was effective collaboration between the different units at MBRU.	☐	☐	☐	☐	☐
c.	Existing processes and procedures were successfully adjusted to meet the challenges of the COVID-19 pandemic.	☐	☐	☐	☐	☐

d.	Innovative solutions were implemented at MBRU in response to the COVID-19 pandemic.	☐	☐	☐	☐	☐
e.	Communication was effective at MBRU during the COVID-19 pandemic.	☐	☐	☐	☐	☐
f.	MBRU's leadership were supportive during the COVID-19 pandemic.	☐	☐	☐	☐	☐
g.	MBRU's staff demonstrated resilience to the changing circumstances of the COVID-19 pandemic.	☐	☐	☐	☐	☐
h.	MBRU's staff demonstrated their willingness to adapt to the changing circumstances of the COVID-19 pandemic.	☐	☐	☐	☐	☐

7. Would you be willing to participate in a semi-structured interview to provide further insight into your experiences and perspectives regarding MBRU's response to the COVID-19 pandemic?

☐Yes, please email the researcher directly to 2020005911@student.sit.ac.nz
☐No

Thank you for your participation in the survey.
