# Supplementary material for: Investigating organizational resilience in a medicine and health sciences university in United Arab Emirates
Source: PLoS One. 2025 Dec 17;20(12):e0338728. doi: 10.1371/journal.pone.0338728 (PMC12711023; doi:10.1371/journal.pone.0338728)
Supplement: S4 File — (PDF) [file pone.0338728.s004.pdf]

Appendix IV: Semi-Structured Interview Protocol

Organisational Resilience at Mohammed Bin Rashid University of Medicine and Health Sciences Interview Questions

1. How prepared do you think MBRU was for the COVID-19 pandemic? Please elaborate on your answer.
2. Please give one to three examples of measures (strategies) that MBRU had in place before the pandemic that enabled its response.

Contributions to success

3. What factors do you think contributed to MBRU’s successful response to the COVID-19 pandemic?
    - **Prompts** -Actions: Digital transformation, crisis planning, collaborations/partnerships, resources, learning and adaptability.
    - **Prompts** -Behaviours: Well-being support, effective leadership, adaptability, effective communication.
    - **Prompts** -Events: Innovation, community engagement.
4. How do you think the organisation’s culture (values) at MBRU influenced its response during the COVID-19 pandemic?

Challenges and adaptability

5. What were the key challenges that MBRU faced during the COVID-19 pandemic?
6. Please give one to three examples of how MBRU adapted to challenges during the COVID-19 pandemic.
7. What aspects of the MBRU’s response to the COVID-19 pandemic could have been better?

Lessons learned, and preparedness for and effects on the future

8. What are the lessons learned (from MBRU’s firsthand experiences with responding to COVID-19) that other higher education institutions/ universities of medicine and health sciences/ academic health systems can benefit from in preparing for future crises?
9. Based on your firsthand experiences with responding to COVID-19, what recommendations would you offer to enhance MBRU's preparedness for future crises?
10. How do you think MBRU’s response to the COVID-19 pandemic affected its development trajectory?
